# Coupling Ni Single Atomic Sites with Metallic Aggregates at Adjacent Geometry on Carbon Support for Efficient Hydrogen Peroxide Electrosynthesis

**DOI:** 10.1002/advs.202402240

**Published:** 2024-04-11

**Authors:** Xin Wang, Run Huang, Xin Mao, Tian Liu, Panjie Guo, Hai Sun, Zhelin Mao, Chao Han, Yarong Zheng, Aijun Du, Jianwei Liu, Yi Jia, Lei Wang

**Affiliations:** ^1^ College of Chemical Engineering Zhejiang University of Technology Hangzhou 310014 P. R. China; ^2^ School of Chemistry Physics and Mechanical Engineering Queensland University of Technology Brisbane QLD 4000 Australia; ^3^ Division of Nanomaterials & Chemistry Hefei National Research Center for Physical Sciences at the Microscale Institute of Energy Hefei Comprehensive National Science Center Department of Chemistry Institute of Biomimetic Materials & Chemistry Anhui Engineering Laboratory of Biomimetic Materials University of Science and Technology of China Hefei 230026 P. R. China; ^4^ Anhui Province Key Laboratory of Advanced Catalytic Materials and Reaction Engineering School of Chemistry and Chemical Engineering Hefei University of Technology Hefei 230041 P. R. China; ^5^ Petroleum and Chemical Industry Key Laboratory of Organic Electrochemical Synthesis College of Chemical Engineering Zhejiang Carbon Neutral Innovation Institute Zhejiang University of Technology (ZJUT) Hangzhou 310014 P. R. China; ^6^ Moganshan Institute ZJUT Deqing 313200 P. R. China

**Keywords:** heteroatom doped carbon matrix, hydrogen peroxide electrosynthesis, nanoparticles, single atoms, synergistic effect

## Abstract

Single atomic catalysts have shown great potential in efficiently electro‐converting O_2_ to H_2_O_2_ with high selectivity. However, the impact of coordination environment and introduction of extra metallic aggregates on catalytic performance still remains unclear. Herein, first a series of carbon‐based catalysts with embedded coupling Ni single atomic sites and corresponding metallic nanoparticles at adjacent geometry is synthesized. Careful performance evaluation reveals Ni_SA_/Ni_NP_‐NSCNT catalyst with precisely controlled active centers of synergetic adjacent Ni‐N_4_S single sites and crystalline Ni nanoparticles exhibits a high H_2_O_2_ selectivity over 92.7% within a wide potential range (maximum selectivity can reach 98.4%). Theoretical studies uncover that spatially coupling single atomic NiN_4_S sites with metallic Ni aggregates in close proximity can optimize the adsorption behavior of key intermediates ^*^OOH to achieve a nearly ideal binding strength, which thus affording a kinetically favorable pathway for H_2_O_2_ production. This strategy of manipulating the interaction between single atoms and metallic aggregates offers a promising direction to design new high‐performance catalysts for practical H_2_O_2_ electrosynthesis.

## Graphical abstract

A new type of electrocatalyst with precisely controlled active centers of synergetic adjacent Ni─N_4_S single sites and crystalline Ni nanoparticles exhibits a high H_2_O_2_ selectivity over 92.7% within a wide potential range (maximum selectivity can reach 98.4%) by oxygen reduction reaction, which demonstrates an excellent candidate for practical H_2_O_2_ electrochemical synthesis.

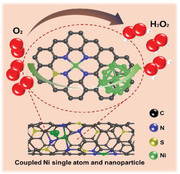

## 1 Introduction

Hydrogen peroxide (H_2_O_2_), ranking among the 100 most essential worldwide chemicals, has been widely used as an eco‐friendly oxidant in broad industries, including wastewater treatment, pulp/paper bleaching, chemical synthesis, medical disinfection, and so on.^[^ ^1^ ^]^ The global market size of H_2_O_2_ was valued at US$ 3.2 billion in 2022 and is predicted to expand rapidly in the near future, achieving US$ 4.1 billion by 2028.^[^ ^2^ ^]^ Until now, nearly all commercial H_2_O_2_ is manufactured industrially through the energy‐consuming anthraquinone oxidation process.^[^ ^3^ ^]^ In fact, this centralized production method needs enormous initial‐investment for infrastructure and easily causes waste pollutants discharge problems.^[^ ^4^ ^]^ Alternatively, direct use of O_2_ and H_2_ as reactants toward H_2_O_2_ synthesis can be realized by a one‐step thermal catalytic route, which, however, poses a significant explosion risk during operation.^[^ ^5^ ^]^

Since the first commercialization of electrochemical on‐site H_2_O_2_ production called the Dow–Huron process realized in 1991, this “green”, cost‐effective, and safe route driven by renewable electricity has attracted numerous attention.^[^ ^6^ ^]^ The critical point of this promising technology is how to reduce O_2_ at the cathode through a 2e^−^ oxygen reduction reaction (ORR) process with high selectivity, because, in most circumstances, the competitive 4e^−^ ORR pathway is hard to suppress, which compromises the H_2_O_2_ yield.^[^ ^7^ ^]^ According to previous theoretical studies, 2e^−^ ORR performance could be estimated using the Sabatier volcano‐type relationship on the ^*^OOH binding energy.^[^ ^8^ ^]^ Therefore, efficient catalysts with the optimal adsorption energy of ^*^OOH closest to the volcano apex are crucial to enable practical H_2_O_2_ electrochemical synthesis.

Carbon‐supported single‐atom catalysts (SACs) have emerged as the research hotspot in electrocatalysis due to their tunable active site configuration of the collaborative metal centers and surrounding atoms.^[^ ^9^ ^]^ Over the past decades, classical nitrogen‐coordinated SACs have been demonstrated to be efficient in 4e^−^ ORR because of the strong binding ability of ^*^OOH.^[^ ^10^ ^]^ To improve 2e^−^ selectivity for H_2_O_2_ generation, recent work disclosed that introducing oxygen atoms to directly or indirectly interact with the metal center could weaken ^*^OOH adsorption and suppress its dissociation.^[^ ^11^ ^]^ By fine‐tuning the non‐metal atomic coordination environment, various highly‐efficient SACs toward H_2_O_2_ electrosynthesis have been developed, such as Fe‐CNT,^[^ ^12^ ^]^ Co_1_‐NG(O),^[^ ^13^ ^]^ Ni‐N_2_O_2_,^[^ ^14^ ^]^ and others.^[^ ^15^ ^]^ Generally, synthesis of these SACs requires high‐temperature treatment and such harsh condition is prone to produce large metal aggregates, like nanoparticles.^[^ ^16^ ^]^ For SACs, residual nanoparticles are commonly regarded as useless species, which are removed by an extra post‐acid‐leaching procedure.^[^ ^17^ ^]^ However, in recent studies, the role of the atomic metal center and surrounding nanoparticle as a synergetic integrity to promote electrocatalytic processes has been confirmed, such as 4e^−^ ORR and hydrogen evolution reaction (HER).^[^ ^18^ ^]^ Moreover, doping nonmetal heteroatoms (S, P, B, Se, etc.) has also manifested its possibility in finely tuning the electronic structure of the metal‐N atomic site.^[^ ^19^ ^]^ Inspired by these studies, it can be reasonably deduced that doped heteroatoms and adjacent nanoparticles may synergistically induce the charge redistribution on single atomic metal‐N sites, which consequently weakens the adsorption energy of ^*^OOH to realize highly‐efficient electrochemical synthesis of H_2_O_2_. However, how to construct such a collaborative structure remains a great challenge at present and the exact synergetic mechanism needs to be clarified.

Herein, we report a highly selective and efficient electrocatalyst that consists of coupling single atomic Ni sites and adjacent metallic Ni confined in the carbon matrix, defined as Ni_SA_/_NP_‐NSCNT. This material with two types of synergetic Ni‐containing sites demonstrates remarkable electrocatalytic performance toward the 2e^−^ ORR for H_2_O_2_ production, with a high selectivity over 92.7% within a wide potential range. Density functional theory (DFT) calculations reveal the synergistically positive function of constructing spatially adjacent configuration comprised with NiN_4_S single atom and metallic Ni_4_ species toward 2e^−^ ORR, which regulates the binding energy of key intermediates ^*^OOH into a proper level of neither too strong or too weak, consequently boosting the catalytic performance of H_2_O_2_ electrosynthesis.

## 2 Results and Discussion

### 2.1 Morphology and Structural Characterizations

**Figure 1:**
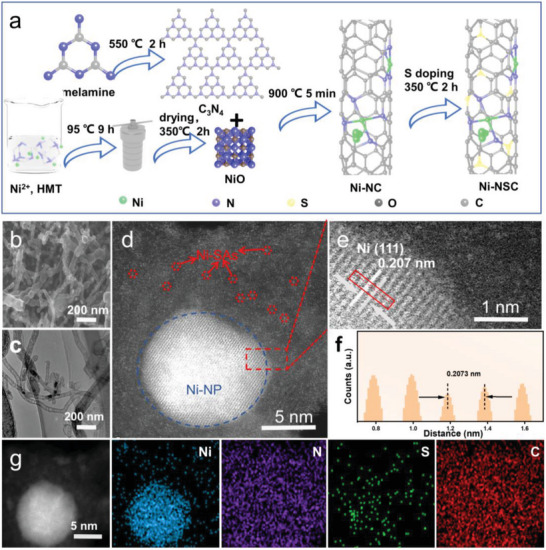
a) Schematic diagram of the synthesis process of Ni_SA_/Ni_NP_‐NSCNT. b) SEM image, c) TEM image, and d) atomic‐resolution HAADF‐STEM image of Ni_SA_/Ni_NP_‐NSCNT, showing both Ni nanoparticles (Ni‐NP, blue dash circle) and Ni single atoms (Ni‐SAs, red dash circles). e) The enlarged HAADF‐STEM image of the red dash box marked the local area of Ni─NP. f) The intensity profile along the line. g) Elemental mapping of Ni_SA_/Ni_NP_‐NSCNT. Ni (blue), N (purple), S (green), C (red).

The Ni_SA_/Ni_NP_‐NSCNT was synthesized via the procedure as shown in **Figure** **1**a, and the details were available in Supporting Information. In brief, C_3_N_4_, a conjugated polymer, was first fabricated via calcination of melamine at 550 °C for 2 h under N_2_ atmosphere (Figure S1, Supporting Information). At the same time, NiO nanosheets were fabricated by a two‐step process (Figure S2, Supporting Information): 1) Ni^2+^ and hexamine (HMT) dissolved in ultrapure water reacted to form Ni‐HMT coordination framework,^[^ ^20^ ^]^ 2) Calcination of Ni‐HMT at 350 °C for 2 h in a muffle furnace. Then, these two materials (C_3_N_4_ and NiO) were mixed homogeneously with a mass ratio of 100:1 and pyrolyzed at 900 °C for 2 h under an inert atmosphere, resulting in Ni‐containing N‐doped carbon materials, called as Ni_SA_/Ni_NP_‐NCNT. Finally, doping sulfur element into Ni_SA_/Ni_NP_‐NCNT yielded the target Ni_SA_/Ni_NP_‐NSCNT. By varying the type of doping elements, such as P and B, other contrast samples were also obtained (Ni_SA_/Ni_NP_‐NPCNT and Ni_SA_/Ni_NP_‐NBCNT).

Scanning electron microscope (SEM) and transmission electron microscope (TEM) images clearly show that Ni_SA_/Ni_NP_‐NSCNT comprises bamboo‐like carbon nanotubes with a diameter of 50–100 nm (Figure 1b,c). Within these nanotubes, small nanoparticles are distributed randomly at the tips or inside (Figure S3, Supporting Information). The aberration‐corrected high‐angle annular dark‐field scanning transmission electron microscopy (AC HAADF‐STEM) was employed to directly observe the atomic structure of Ni_SA_/Ni_NP_‐NSCNT. The atomic‐resolution HAADF‐ STEM image in Figure 1d confirms the coexistence of Ni single atoms (Ni SAs, highly dispersed bright spots marked with red dash circles) and Ni nanoparticles (Ni NP, marked with a blue dash circle) throughout the carbon matrix. The magnified image and corresponding intensity profile along the marked line clearly reveal the well‐crystallized Ni NP with a lattice distance of 0.207 nm, which coincides with the (111) planes of Ni (Figure 1e,f). In addition, some bright spots representing the Ni SAs are also found to be located closely surrounding the Ni NP with the spatial distance of less than 1 nm, demonstrating the successful construction of strongly interacted multi‐sites that are composed of Ni NP and satellite SAs. Such short inter‐site distance should facilitate the electron transfer between NP and adjacent SAs, thus benefiting the catalytic process.^[^ ^21^ ^]^ The elemental mapping suggests the uniform distribution of Ni, C, N, and S in Ni_SA_/Ni_NP_‐NSCNT. Notably, the presence of Ni NP surrounded by abundant Ni SAs is noticeable, but the signal of the S element is weak due to low doping content.

The morphology and microstructure of Ni_SA_/Ni_NP_‐NCNT, Ni_SA_/Ni_NP_‐NPCNT, and Ni_SA_/Ni_NP_‐NBCNT were also examined, which reveal similar bamboo‐like carbon nanotubes with embedded Ni NP and abundant SAs (Figures S4–S6, Supporting Information). As known to all, treatment temperature significantly affects the final state and structure of produced carbon materials.^[^ ^22^ ^]^ The thermogravimetric‐differential scanning calorimetry (TG‐DSC) analysis reveals that the mass loss step of C_3_N_4_/NiO mixture during the pyrolysis ends almost over 700 °C, which yields ≈28% of products (Figure S7, Supporting Information). Therefore, annealing temperature of C_3_N_4_/NiO mixture was adjusted within the range of 800 to 1000 °C to identify the best reaction conditions. Apparently, SEM images in (Figure S8, Supporting Information)indicate that a lower temperature of 800 °C poses negligible influence on the morphology while increasing the temperature to 1000 °C can destroy the nanotubes to form architectures composed of interconnected frazzles with a less porous feature. Previous studies have uncovered that the catalytic activity of electrocatalysts is highly correlated with electronic conductivity and pore structure.^[^ ^23^ ^]^ Accordingly, considering the structural integrity and electrical property of as‐made carbon‐based materials, 900 °C is set to be the optimal treatment temperature for the pyrolysis process of mixed C_3_N_4_/NiO powders. Without special emphasis, the samples mentioned below are all prepared through a pyrolysis process at 900 °C followed by corresponding heteroatoms doping.

**Figure 2:**
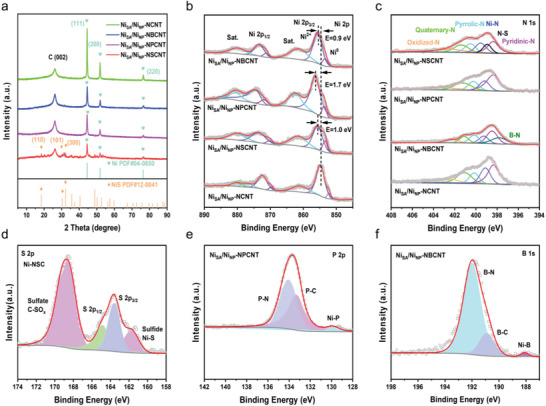
a) PXRD patterns, b) Ni 2p XPS spectra, and c) N 1s XPS spectra of Ni_SA_/Ni_NP_‐NCNT, Ni_SA_/Ni_NP_‐NBCNT, Ni_SA_/Ni_NP_‐NPCNT and Ni_SA_/Ni_NP_‐NSCNT. d) S 2p XPS spectrum of Ni_SA_/Ni_NP_‐NSCNT. e) P 2p XPS spectrum of Ni_SA_/Ni_NP_‐NPCNT. f) B 1s XPS spectrum of Ni_SA_/Ni_NP_‐NBCNT.

The power X‐ray diffraction (PXRD) patterns confirm the Ni NPs in all four synthesized catalysts (Ni_SA_/Ni_NP_‐NCNT, Ni_SA_/Ni_NP_‐NBCNT, Ni_SA_/Ni_NP_‐NPCNT and Ni_SA_/Ni_NP_‐NSCNT) (**Figure** **2**a). In addition to the broad peak at around 26°, which is associated with the (002) plane of graphitic carbon, there are distinct characteristic diffraction peaks of metallic Ni (JCPDS: 04–0850) located at 45°, 52° and 76°, corresponding to the (111), (200), and (220) facets, respectively. It is noteworthy that, for Ni_SA_/Ni_NP_‐NSCNT, weak peaks belonging to crystalline NiS (JCPDS: 12–0014) can also observed, indicating the formation of a few NiS nanocrystals when incorporating S dopants due to strong interaction and nucleation tendency between transition metal and S atoms.^[^ ^24^ ^]^ In order to investigate the contribution of residue NiS nanoparticles in Ni_SA_/Ni_NP_‐NSCNT toward electrocatalytic performance, we also prepared a reference sample that resembled the structure of Ni_SA_/Ni_NP_‐NSCNT except without possessing NiS nanocrystals, denoted as R‐Ni_SA_/Ni_NP_‐NSCNT (Figure S9, Supporting Information). Cyclic voltammetry (CV) test in 0.1 m KOH solutions under H_2_‐saturated condition with a Pt‐foil as the working electrode shows the RHE calibration equation is E(RHE) = E(Ag/AgCl) + 0.960 (Figure S10, Supporting Information).^[^ ^25^ ^]^ ORR testing clearly demonstrates that eliminating NiS species has little impact on the catalytic activity for H_2_O_2_ electrosynthesis (Figure S11, Supporting Information), which is in accordance with previous reports that nickel sulfide cannot effectively catalyze the ORR process as other superior catalysts.^[^ ^26^ ^]^ The Raman spectra of as‐made four samples exhibit two prominent peaks at 1350 and 1590 cm^−1^, corresponding to the D band (defects) and G band (graphitic sp^2^ carbon) of carbon species, respectively (Figure S12, Supporting Information).^[^ ^10b^ ^]^ The similar value of I_D_/I_G_ ratios indicate that introducing secondary heteroatoms (P, S, or B) leads to negligible influence on the carbon structures, and the key factor to determine the catalytic performance should mainly lie on the difference of coordination environments around active metallic centers. The N_2_ adsorption/desorption curve of Ni_SA_/Ni_NP_‐NSCNT display a typical type‐IV physisorption isotherm, implying its porous nature, which can increase the active sites exposure and enhance mass transport process during catalysis (Figure S13, Supporting Information).^[^ ^27^ ^]^

X‐ray photoelectron spectroscopy (XPS) was conducted to study the chemical state of elements over the catalyst surface. The XPS survey spectra show obvious peaks of Ni, N, C, and O and corresponding doping elements (S, P or B) in these samples (Figure S14, Supporting Information). The high‐resolution Ni 2p XPS spectra in Figure 2b can all be well‐deconvoluted into several sub‐peaks corresponding to metallic Ni^0^ (852.5–853.0 eV), Ni^2+^ (≈855.7 eV) and two related satellite peaks.^[^ ^28^ ^]^ A noticeable shift of Ni^2+^ peak for Ni_SA_/Ni_NP_‐NBCNT, Ni_SA_/Ni_NP_‐NPCNT, and Ni_SA_/Ni_NP_‐NSCNT as compared with that in Ni_SA_/Ni_NP_‐NCNT can be observed, suggesting a change in the coordination environment surrounding Ni SAs upon doping additional heteroatoms (B, P, S). As demonstrated previously, the electrocatalytic performance of SACs is closely related to the specific metal species and their local atomic environments. Heteroatom doping of traditional metal‐N_4_ single atom sites with other non‐metal elements such as S, P, and B can change the local coordination structure of the center metal site, encompassing the first and second coordination sphere, which thus leads to the variation of the electronic state.^[^ ^19^, ^29^ ^]^ For ORR, this may optimize the adsorption/desorption process of oxygen‐related intermediates, consequently boosting the electrocatalytic activity. The high‐resolution N 1s XPS spectra in Figure 2c present five or six fitted characteristic sub‐peaks, including N‐B bond (397.9 eV), pyridinic N (398.5 eV), N─S bond (398.9 eV), Ni─N (399.2 eV), pyrrolic N (400.1 eV), graphitic N (401.1 eV) and oxidized N (402.3 eV).^[^ ^18^, ^30^ ^]^ This confirms the successful incorporation of N into the carbon matrix, with some directly bonded with Ni atoms to form Ni─N moieties. The intensity change of deconvoluted peaks is caused by the secondary doped S, P, or B bonding with N atoms.^[^ ^16^, ^31^ ^]^ The C 1s XPS spectra can be divided into four pronounced peaks ascribed to C─C (graphitic sp^2^ carbon, 284.6 eV), C─‐N (285.3 eV), C─O (287.3 eV), and C═O (289.3 eV) species, respectively (Figure S15, Supporting Information).^[^ ^18^, ^32^ ^]^ The binding energy shift of these characteristic peaks in a series of synthesized samples should also originate from the difference of doping configurations in carbon lattice. Figure 2d shows the S 2p XPS spectrum of Ni_SA_/Ni_NP_‐NSCNT, which reveals C‐SO_x_ (168.7 eV), C─S─C (two peaks located at 163.7 and 164.9 eV), and Ni─S (161.9 eV) coordination,^[^ ^31^, ^33^ ^]^ and the content of NiS increases slightly at higher temperatures (Figure S16, Supporting Information). The presence of Ni─S peak can be attributed to the formation of a few NiS nanocrystals, which has been demonstrated in the aforementioned XRD results. S dopant in the carbon matrix is supported by the existence of C─S─C peak. For C─SO_x_ bond, electron‐withdrawing effect on a carbon plane has been verified in previous reports to enable a beneficial electronic structure toward electrocatalysis.^[^ ^24^, ^34^ ^]^ As NiS has been verified to be useless for electrocatalysis in Ni_SA_/Ni_NP_‐NSCNT, we further analyze the S 2p XPS spectrum of R‐Ni_SA_/Ni_NP_‐NSCNT (Figure S17, Supporting Information). It can be found that the characteristic peak of Ni─S bond disappears, leaving only three fitted peaks assigned to C─SO_x_ and C─S─C. This indicates S dopants in the true active moieties of Ni_SA_/Ni_NP_‐NSCNT are not directly bonded to the centered Ni SAs. Further considering the existence of N─S bond in the corresponding N 1s XPS spectrum, the S atoms in Ni_SA_/Ni_NP_‐NSCNT may locate at the second coordination shell of a single Ni site to form a bond with the N atom at the first coordination shell. The P 2p spectrum of Ni_SA_/Ni_NP_‐NPCNT displays three fitted peaks centered at 130.1, 133.2, and 134.1 eV, which are attributed to Ni─P, P─C and P─N bonds, respectively Figure 2e.^[^ ^35^ ^]^ As the temperature rises, the peak of Ni─P progressively expands, possibly due to the increase in the specific gravity of defective carbon (known as N high‐temperature spillover) favoring P doping (Figures S12 and S16, Supporting Information). In the high‐resolution B 1s XPS spectrum of Ni_SA_/Ni_NP_‐NBCNT, beside the two dominant peaks corresponding to B─C (191.0 eV) and B─N (192.1 eV), an additional small peak located at 188.2 eV can also be visualized, representing the Ni─B bond Figure 2f.^[^ ^36^ ^]^ To further elucidate the impact of acid post‐treatment on the structure of all samples, we also synthesized R‐Ni_SA_/Ni_NP_‐NPCNT and R‐Ni_SA_/Ni_NP_‐NBCNT in addition to R‐Ni_SA_/Ni_NP_‐NSCNT. Negligible change in the XPS spectra can be observed in comparison with the samples before acidic etching with HCl (Figures S18 and S19, Supporting Information), still maintaining the original Ni─P/B peaks, which indicates the resilience of single atomic sites to withstand acid‐treatment. Based on these results, we can speculate that the S atom may be located in the second coordination sphere around the atomic Ni center in Ni_SA_/Ni_NP_‐NSCNT,^[^ ^30b^ ^]^ while P and B atoms serve as the direct bonding sites with the Ni center by partial replacing N atoms of first coordination sphere in Ni_SA_/Ni_NP_‐NPCNT^[^ ^35^ ^]^ and Ni_SA_/Ni_NP_‐NBCNT.^[^ ^37^ ^]^

**Figure 3:**
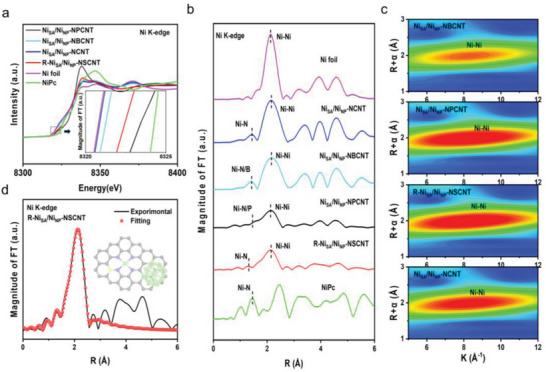
a) Ni K‐edge XANES spectra and b) Fourier transformation of EXAFS spectra of R‐Ni_SA_/Ni_NP_‐NSCNT and reference materials. The inset in Figure 3a shows the enlarged boxed area. c) WTs of the EXAFS spectra. d) EXAFS fitting curves of R‐Ni_SA_/Ni_NP_‐NSCNT.

To further identify the local coordination environment of Ni in the catalysts, X‐ray absorption near‐edge structure (XANES) and extended X‐ray absorption fine structure (EXAFS) measurements were carried out. **Figure** **3**a shows the XANES spectra at the Ni K edge of R‐Ni_SA_/Ni_NP_‐NSCNT and reference materials. The adsorption edges of all synthesized samples are located between those of Ni foil and NiPc, suggesting the Ni valence state is between 0 and +2, consistent with TEM and XPS results. Fourier‐transformed EXAFS curves of Ni_SA_/Ni_NP_‐NCNT, Ni_SA_/Ni_NP_‐NBCNT, Ni_SA_/Ni_NP_‐NPCNT, and R‐Ni_SA_/Ni_NP_‐NSCNT reveal a dominant Ni─Ni contribution with the peak at ≈2.1 Å in R space, confirming the formation of Ni NPs in our‐synthesized catalysts. Moreover, weak signals ranging from 1.4 to 1.6 Å can also be detected, quite close to Ni─N peak of NiPc, which corroborates the existence of isolated Ni atoms bonded with nonmetal atoms (Ni─NM coordination) (Figure 3b). The wavelet transform (WT)‐EXAFS technique is a powerful tool to distinguish the backscattering atoms in both the R‐space and k‐space. As shown in (Figure 3c; Figure S20, Supporting Information), the WT contour plots of as‐prepared different samples exhibit the maximum intensity at ≈8.0 Å^−1^, which can be obviously distinguished from NiPc (6.3 Å^−1^) and Ni foil (8.5 Å^−1^), further affirming the co‐presence of atomically dispersed Ni sites and Ni nanocrystals.^[^ ^21^, ^24^ ^]^ Finally, the quantitative EXAFS fitting was conducted to precisely determine the local coordination environment of Ni species. According to fitting results in (Figure 3d; Figures S21 and S22; Table S1, Supporting Information), the coordination numbers of Ni─N and Ni─Ni in R‐Ni_SA_/Ni_NP_‐NSCNT are ≈3.83 and 1.75, respectively, indicating both Ni NPs and NiN_4_ single sites are formed. Particularly, the fitting data shows the position of the S atom does not located at the first coordination shell. Therefore, based on the XPS and XAFS results, NiN_4_S single atomic site (S locates at the second coordination sphere of center Ni site) accompanied with Ni_4_ clusters in carbon matrix are constructed to represent the active structures of R‐Ni_SA_/Ni_NP_‐NSCNT and typical Ni_SA_/Ni_NP_‐NSCNT (contribution of residual little NiS nanocrystals in Ni_SA_/Ni_NP_‐NSCNT can be neglected according to aforementioned analysis), which will be used in the following DFT calculations. For contrast, Ni_SA_/Ni_NP_‐NCNT, Ni_SA_/Ni_NP_‐NBCNT, and Ni_SA_/Ni_NP_‐NPCNT exhibit similar atomic coordination structures as that of R‐Ni_SA_/Ni_NP_‐NSCNT, also containing both Ni NPs and SAs (Coordination number: ≈4; Fitted coordination configuration: NiN_4_, NiN_3_B, and NiN_3_P, respectively).

### 2.2 Performance Testing

**Figure 4:**
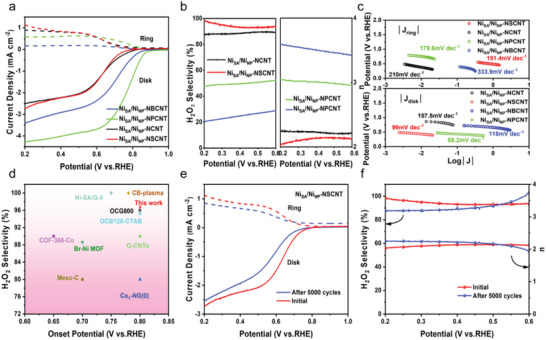
a) Polarization curves of Ni_SA_/Ni_NP_‐NSCNT, Ni_SA_/Ni_NP_‐NPCNT, Ni_SA_/Ni_NP_‐NBCNT and Ni_SA_/Ni_NP_‐NCNT on RRDE at 1600 rpm in O_2_‐saturated 0.1 m KOH, b) The calculated H_2_O_2_ selectivity and Transfer electron number (n) of catalysts, c) Tafel slope of Ni_SA_/Ni_NP_‐NSCNT, Ni_SA_/Ni_NP_‐NPCNT, Ni_SA_/Ni_NP_ ‐NBCNT, Ni_SA_/Ni_NP_‐NCNT, d) Ni_SA_/Ni_NP_‐NSCNT and 2e‐ ORR performance of various reported electrocatalysts. e) and f) Stability test of Ni_SA_/Ni_NP_‐NSCNT catalyst after 5000 cycles.

The ORR performance of the catalysts was evaluated using a rotating ring disc electrode (RRDE) on a three‐electrode system in 0.1 m KOH solution. All potentials were calibrated to the reversible hydrogen electrode (RHE), the rotational speed was kept at 1600 rpm for the duration of the test, and the platinum ring potential was set to 1.2 V versus RHE to quantify the H_2_O_2_ produced at the disc electrode. Linear sweep voltammetry (LSV) curves in **Figure** **4**a reveal that Ni_SA_/Ni_NP_‐NSCNT displays limited disk current density of ≈2.78 mA cm^−2^, which is closest to the theoretical mass transport limit (≈3 mA cm−2) of the 2e− ORR process among all the tested samples.^[^ ^1^, ^38^ ^]^ The onset potential (defined as the potential at the current density of 0.1 mA cm^−2^) of Ni_SA_/Ni_NP_‐NCNT and Ni_SA_/Ni_NP_‐NSCNT are both located at around 0.8 V, slightly higher than the thermodynamic limit of 0.76 V (in 0.1 m KOH, pH 13), in consistence with previous literatures that low H_2_O_2_ concentration in alkaline media and high 2e^−^ ORR catalytic activity can lead to a Nernst‐related potential shift beyond the limit.^[^ ^7^, ^13^, ^39^ ^]^ For Ni_SA_/Ni_NP_‐NPCNT and Ni_SA_/Ni_NP_‐NBCNT, more positive onset potential (≈0.95 and 0.9 V), higher limited disk current density (≈4.25 and 3.4 mA cm^−2^), and lower ring current density (≈0.6 and 0.2 mA cm^−2^) can be observed, which imply their favorable dynamics for 4e^−^ ORR pathway. As shown in Figure 4b, most electrons are consumed by the 2e^−^ pathway on Ni_SA_/Ni_NP_‐NCNT and Ni_SA_/Ni_NP_‐NSCNT over a wide potential range, delivering a high H_2_O_2_ FE of more than 90%. Significantly, Ni_SA_/Ni_NP_‐NSCNT achieves the highest performance in H_2_O_2_ electrosynthesis from ORR, with a maximum FE (≈98.4%) and electron transfer numbers closest to 2 (≈2.03). In contrast, the worst electrocatalytic activities of H_2_O_2_ production from ORR among all the tested samples can be found on Ni_SA_/Ni_NP_‐NBCNT, with only 30% of H_2_O_2_ selectivity and high electron transfer numbers close to 4 (≈3.5). The low 2e^−^ ORR performance of Ni_SA_/Ni_NP_‐NBCNT can be attributed to two main factors. First, during B doping, a significant number of B─N bonds are formed, which may lead to the adjustment of the binding energy of the ^*^OOH intermediate, consequently promoting the 4e‐ ORR.^[^ ^30c^ ^]^ Second, the introduced B atoms cause a direct alteration in the geometry of the first coordination site, which in turn impacts the electronic properties of the Ni active center. This modification subsequently influences the adsorption/desorption behavior of ORR intermediates, ultimately facilitating the 4e^−^ ORR kinetics.^[^ ^37^ ^]^

The Tafel plots were calculated to analyze the reaction kinetics and rate‐determining steps (RDS) (Figure 4c). The slopes of various samples from the disk electrode are 157.5 (Ni_SA_/Ni_NP_‐NCNT), 118 (Ni_SA_/Ni_NP_‐NBCNT), 99 (Ni_SA_/Ni_NP_‐NSCNT) and 68.2 (Ni_SA_/Ni_NP_‐NPCNT) mV dec^−1^, respectively, validating a change in RDS from the first ^*^OOH formation step (O_2_ → ^*^OOH, ≈120 mV dec^−1^) to the subsequent dissociation step (^*^OOH → H_2_O_2_, ≈ 70 mV dec^−1^).^[^ ^40^ ^]^ Moreover, Ni_SA_/Ni_NP_‐NSCNT displays the lowest slope at the ring electrode compared to catalysts, suggesting the most rapid H_2_O_2_ production kinetics, which may be attributed the optimized electronic structure and associated adsorption/desorption process.^[^ ^41^ ^]^ To the best of our knowledge, Ni_SA_/Ni_NP_‐NSCNT can realize the high H_2_O_2_ molar selectivity over 90% within a wide potential window of 0.2–0.6 V, which overwhelms many reported catalysts for H_2_O_2_ electroproduction (Figure 4d; Table S2, Supporting Information).

The catalyst stability was then investigated by accelerated degradation test (ADT) analysis, displayed in Figure 4e,f. The LSV curves show a slight negative shift after 5000 cycles of ADT operation, but high H_2_O_2_ selectivity with ≈87% is still maintained, confirming the superb durability of Ni_SA_/Ni_NP_‐NSCNT for H_2_O_2_ electrosynthesis. After the ADT stability test, the Ni_SA_/Ni_NP_‐NSCNT still retains its initial structure with both nanoparticles and surrounding single atoms embedded in nanotubes, as shown in (Figure S23, Supporting Information). The absence of detectable Ni elements in the electrolyte, as indicated by the inductively coupled plasma‐optical emission spectrometry (ICP‐OES) results, suggests that metal‐leaching does not occur during the ORR process. Aiming to uncover the reason of the slight activity degradation for post‐tested Ni_SA_/Ni_NP_‐NSCNT, XPS analysis was further conducted to probe the chemical state, which revealed a minor negative shift in the binding energy of Ni^2+^ species as compared with the original sample (Figure S23d, Supporting Information). This shift suggests an accumulation of more electrons at the active Ni sites during 2e^−^ ORR process. According to early studies, for ORR electrocatalysis, the increase of charge density at the active centers can promote the adsorption ability of oxygen‐related species, thus boosting the 4e^−^ pathway.^[^ ^16^, ^42^ ^]^ In this work, such change in electronic structure can explain why H_2_O_2_ selectivity of Ni_SA_/Ni_NP_‐NSCNT is slightly decreased after long‐term operation. To evaluate the possibility for industrial application in a real device, we prepared our catalyst on carbon paper and tested it in a custom‐made electrochemical H‐type cell (Figure S24, Supporting Information). Encouragingly, the H_2_O_2_ productivity of Ni_SA_/Ni_NP_‐NSCNT can reach 422.5 mmol g^−1^ h^−1^ (0.66 V vs RHE) under an alkaline condition, which demonstrates the feasibility of electrochemical H_2_O_2_ production for our catalysts.

To further confirm the synergistic effect of Ni single atoms and Ni nanoparticles in the electrosynthesis of H_2_O_2_, two key contrast experiments were conducted. First, single‐atom poisoning experiments were carried out to evaluate the role of Ni nanoparticles as active sites alone, as shown in (Figure S25a,b, Supporting Information). The findings clearly demonstrated a significant shift in the ORR pathway from 2e^−^ to 4e^−^ upon the introduction of KSCN as a single‐atom chelant. This transformation underscored the crucial contribution of Ni single‐atomic sites within Ni_SA_/Ni_NP_‐NSCNT toward enhancing H_2_O_2_ electrosynthesis efficiency. Subsequently, to explore the catalytic capabilities of Ni single atoms alone in facilitating the 2e^−^ ORR, a reference sample comprising solely NiN_4_S single‐atomic sites, denoted as Ni‐NSC, was prepared based on previous research.^[^ ^43^ ^]^ Electrochemical evaluations of Ni‐NSC exhibited a H_2_O_2_ selectivity range of 60–70%, which although lower than the typical Ni_SA_/Ni_NP_‐NSCNT, surpassed the performance of samples featuring only Ni_NP_ active centers in the poisoning experiment, as illustrated in (Figure S25c,d, Supporting Information). These results suggest that while Ni single atoms can catalyze the 2e^−^ ORR process within Ni_SA_/Ni_NP_‐NSCNT, the presence of Ni nanoparticles remains crucial in serving as synergists to enhance ORR electrocatalysis for H_2_O_2_ production on the Ni single‐atomic sites.

### 2.3 DFT Calculations

**Figure 5:**
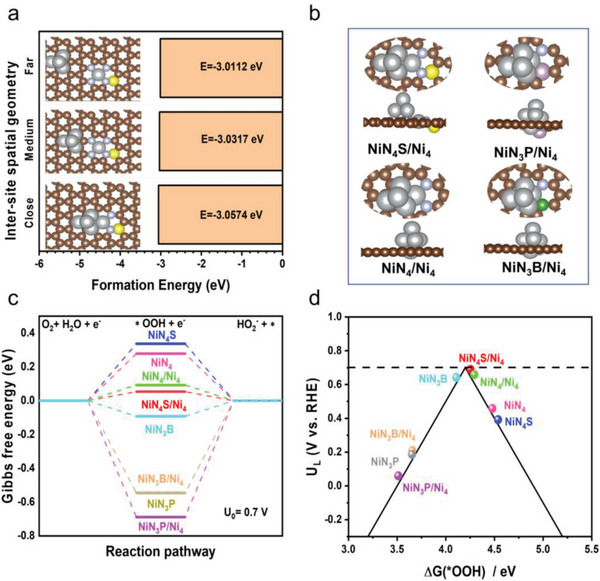
a) The calculated formation energies of models composed of synergetic Ni single atom (NiN_4_S) and metallic Ni (Ni_4_) with different spacing distances (close, medium, and far). b) The optimized structural models that simulate experimental results of as‐prepared samples, including NiN_4_S/Ni_4_, NiN_3_P/Ni_4_, NiN_4_/Ni_4_ and NiN_3_B/Ni_4_. Color code: the brown, gray, yellow, green, blue, and purple balls refer to C, Ni, S, B, N, and P atoms, respectively. c) The calculated Gibbs free‐energy diagram for oxygen reduction to HO_2_^−^ at pH 13.0. d) The calculated volcano plot of the labeled sites toward H_2_O_2_ production. The UL is plotted as a function of ΔG^*^OOH.

DFT calculations were conducted to predict the structural stability of NiN_4_S/Ni_4_ synergistic models with different spacing distances (close, medium, and far) and 2e^−^ ORR energetics of optimized stable models, including isolated single Ni atoms (Figure S26, Supporting Information, NiN_4_, NiN_4_S, NiN_3_P and NiN_3_B) and SA/NP coupling configurations (NiN_4_S/Ni_4_, NiN_3_P/Ni_4_, NiN_4_/Ni_4_ and NiN_3_B/Ni_4_). According to previous reports, the interactions between a single atomic site and corresponding aggregates (cluster or nanoparticle) on a carbon matrix are strongly related with their spacing distance, which consequently impacts the overall catalytic performance of catalysts.^[^ ^18^, ^44^ ^]^ Therefore, the formation energies of NiN_4_S/Ni_4_ coupling models with diverse inter‐site distance were calculated and compared to achieve the most stable structural configuration. As shown in **Figure** **5**a, the adjacent geometry of the two coupling sites, which possesses the smallest inter‐site distance, exhibits the lowest formation energy with ≈ −3.06 eV, suggesting Ni single atom and Ni_4_ species (representing metallic Ni) prefer locating in close proximity to each other on carbon support when forming synergistic active sites. Base on this, a series of SA/NP coupling models with the nearest inter‐site spatial distance were constructed, displayed in Figure 5b. The free energy diagram in Figure 5c shows that NiN_4_S/Ni_4_ has a negligible uphill energy barrier (0.05 eV) for 2e^−^ ORR under alkaline conditions, which is significantly lower than those of other models, indicating its high activity of H_2_O_2_ electroproduction. In principle, the rate‐determining step (RDS) of 2e^−^ ORR belongs to either O_2_ adsorption or ^*^OOH desorption. Therefore, the adsorption‐free energy of key intermediates ^*^OOH (ΔG_*OOH_) is always used as the key descriptor to predict the 2e^−^ ORR activity.^[^ ^45^ ^]^ Previous theoretical studies have confirmed ideal ΔG_*OOH_ for 2e^−^ ORR is ≈4.2 eV, which leads to the optimal thermodynamic limiting potential (U_L_) close to 0.7 V (the equilibrium potential of 2e^−^ ORR with minimized overpotential).^[^ ^1^, ^46^ ^]^ According to the calculation results, the activity volcano plot was summarized as a function of ΔG_OOH*_ (Figure 5d, Supporting Information). NiN_3_B, NiN_3_B/Ni_4_, NiN_3_P, and NiN_3_P/Ni_4_ situate at the left‐hand side of the volcano plot, suggesting the strong ^*^OOH binding that can benefit the dissociation of O─O bond to facilitate the 4e^−^ ORR toward selective H_2_O formation.^[^ ^47^ ^]^ In contrast, NiN_4_/Ni_4_, NiN_4,_ and NiN_4_S are located at the right‐hand side, implying weak ^*^OOH binding to drive high‐selectivity H_2_O_2_ formation. However, the 2e^−^ ORR activity under such circumstances is still unsatisfactory because the first step by converting O_2_ into ^*^OOH becomes the RDS with a high energy barrier, as shown in Figure 5c. Remarkably, NiN_4_S/Ni_4_ exhibits a moderate ∆G_*OOH_ of 4.22 eV and near‐zero overpotential, which is closest to the vertex of the volcano map, verifying the top activity and selectivity for 2e^−^ ORR with a favorable neither too strong nor too weak ^*^OOH binding. (Figure S27, Supporting Information) presents the ^*^OOH adsorption configurations (directly adsorbed on a single atomic Ni site) on each model. The computational results here provide a deep understanding on the catalytic mechanism of our materials that coupling specific single atomic site with corresponding metallic species at adjacent geometry on carbon matrix can maximize the 2e^−^ ORR performance by optimizing the intermediate's adsorption/desorption behavior.

## 3 Conclusion

In summary, we systematically investigate the synergistic effect of coupling sites consisting of single atomic Ni sites and corresponding adjacent Ni nanoparticles toward 2e^−^ ORR for H_2_O_2_ production. The synergy can be finely regulated via tuning the coordination environment through introducing different nonmetal dopants. The best Ni_SA_/Ni_NP_‐NSCNT catalyst, with precisely controlled active centers of synergetic adjacent Ni‐N_4_S single sites and crystalline Ni nanoparticles, exhibits a high H_2_O_2_ selectivity over 92.7% within a wide potential range and can achieve the maximum FE of 98.4%. Theoretical studies uncover that spatially coupling single atomic NiN_4_S sites with metallic Ni aggregates in close proximity can optimize the adsorption behavior of key intermediates ^*^OOH to give a nearly ideal binding strength. Such optimization affords a kinetically favorable pathway for H_2_O_2_ production. This strategy of manipulating the interaction between single atoms and metallic aggregates offers a promising direction to design new high‐performance catalysts for practical H_2_O_2_ electrosynthesis.

## Conflict of Interest

The authors declare no conflict of interest.

## Supporting information

Supporting Information

## Data Availability

The data that support the findings of this study are available from the corresponding author upon reasonable request.

## References

[1] a) S. Siahrostami , A. Verdaguer‐Casadevall , M. Karamad , D. Deiana , P. Malacrida , B. Wickman , M. Escudero‐Escribano , E. A. Paoli , R. Frydendal , T. W. Hansen , I. Chorkendorff , I. E. L. Stephens , J. Rossmeisl , Nat. Mater. 2013, 12, 1137; 24240242 10.1038/nmat3795

[2] Y.‐R. Zheng , S. Hu , X.‐L. Zhang , H. Ju , Z. Wang , P.‐J. Tan , R. Wu , F.‐Y. Gao , T. Zhuang , X. Zheng , J. Zhu , M.‐R. Gao , S.‐H. Yu , Adv. Mater. 2022, 34, 2205414. 10.1002/adma.202205414 36042002

[3] a) J. M. Campos‐Martin , G. Blanco‐Brieva , J. L. G. Fierro , Angew. Chem., Int. Ed. Engl. 2006, 45, 6962; 17039551 10.1002/anie.200503779

[4] a) S. C. Perry , D. Pangotra , L. Vieira , L.‐I. Csepei , V. Sieber , L. Wang , C. Ponce de León , F. C. Walsh , Nat. Rev. Chem. 2019, 3, 442;

[5] a) S. Siahrostami , Chem. Catal. 2023, 3, 100568;

[6] a) Q. Wu , H. Zou , X. Mao , J. He , Y. Shi , S. Chen , X. Yan , L. Wu , C. Lang , B. Zhang , L. Song , X. Wang , A. Du , Q. Li , Y. Jia , J. Chen , X. Yao , Nat. Commun. 2023, 14, 6275; 37805502 10.1038/s41467-023-41947-7 PMC10560253

[7] a) H. W. Kim , M. B. Ross , N. Kornienko , L. Zhang , J. Guo , P. Yang , B. D. McCloskey , Nat. Catal. 2018, 1, 282;

[8] a) Y. Bu , Y. Wang , G.‐F. Han , Y. Zhao , X. Ge , F. Li , Z. Zhang , Q. Zhong , J.‐B. Baek , Adv. Mater. 2021, 33, 2103266; 10.1002/adma.202103266 34562030

[9] a) Y. Shang , X. Duan , S. Wang , Q. Yue , B. Gao , X. Xu , Chinese Chem. Lett. 2022, 33, 663;

[10] a) L. Jiao , J. Li , L. L. Richard , Q. Sun , T. Stracensky , E. Liu , M. T. Sougrati , Z. Zhao , F. Yang , S. Zhong , H. Xu , S. Mukerjee , Y. Huang , D. A. Cullen , J. H. Park , M. Ferrandon , D. J. Myers , F. Jaouen , Q. Jia , Nat. Mater. 2021, 20, 1385; 34112977 10.1038/s41563-021-01030-2

[11] a) P. Cao , X. Quan , X. Nie , K. Zhao , Y. Liu , S. Chen , H. Yu , J. G. Chen , Nat. Commun. 2023, 14, 172; 36635287 10.1038/s41467-023-35839-z PMC9837053

[12] K. Jiang , S. Back , A. J. Akey , C. Xia , Y. Hu , W. Liang , D. Schaak , E. Stavitski , J. K. Nørskov , S. Siahrostami , H. Wang , Nat. Commun. 2019, 10, 3997. 31488826 10.1038/s41467-019-11992-2 PMC6728328

[13] E. Jung , H. Shin , B.‐H. Lee , V. Efremov , S. Lee , H. S. Lee , J. Kim , W. Hooch Antink , S. Park , K.‐S. Lee , S.‐P. Cho , J. S. Yoo , Y.‐E. Sung , T. Hyeon , Nat. Mater. 2020, 19, 436. 31932671 10.1038/s41563-019-0571-5

[14] Y. Wang , R. Shi , L. Shang , G. I. N. Waterhouse , J. Zhao , Q. Zhang , L. Gu , T. Zhang , Angew. Chem., Int. Ed. Engl. 2020, 59, 13057. 32342611 10.1002/anie.202004841

[15] a) G. Wei , Y. Li , X. Liu , J. Huang , M. Liu , D. Luan , S. Gao , X.‐W. Lou , Angew. Chem., Int. Ed. Engl. 2023, 62, 202313914; 10.1002/anie.202313914 37789565

[16] a) Y. Zhou , R. Lu , X. Tao , Z. Qiu , G. Chen , J. Yang , Y. Zhao , X. Feng , K. Müllen , J. Am. Chem. Soc. 2023, 145, 3647; 36744313 10.1021/jacs.2c12933 PMC9936543

[17] a) X. Wang , Y. Jia , X. Mao , D. Liu , W. He , J. Li , J. Liu , X. Yan , J. Chen , L. Song , A. Du , X. Yao , Adv. Mater. 2020, 32, 2000966; 10.1002/adma.202000966 32134518

[18] a) S.‐N. Zhao , J.‐K. Li , R. Wang , J. Cai , S.‐Q. Zang , Adv. Mater. 2022, 34, 2107291; 10.1002/adma.202107291 34796559

[19] T. Sun , S. Mitchell , J. Li , P. Lyu , X. Wu , J. Pérez‐Ramírez , J. Lu , Adv. Mater. 2020, 33, 2003075. 10.1002/adma.202003075 33283369

[20] a) S. Liu , J. Zhou , H. Song , Adv. Energy Mater. 2018, 8, 1800569;

[21] a) Z. Jin , P. Li , Y. Meng , Z. Fang , D. Xiao , G. Yu , Nat. Catal. 2021, 4, 615;

[22] a) Y. Gao , S. Liang , B. Liu , C. Jiang , C. Xu , X. Zhang , P. Liang , M. Elimelech , X. Huang , Nat. Commun. 2023, 14, 2059; 37045829 10.1038/s41467-023-37676-6 PMC10097648

[23] a) L. Peng , J. Yang , Y. Yang , F. Qian , Q. Wang , D. Sun‐Waterhouse , L. Shang , T. Zhang , G. I. N. Waterhouse , Adv. Mater. 2022, 34, 2202544; 10.1002/adma.202202544 35584394

[24] J. Chen , B. Huang , R. Cao , L. Li , X. Tang , B. Wu , Y. Wu , T. Hu , K. Yuan , Y. Chen , Adv. Funct. Mater. 2022, 33, 2209315.

[25] Z. Zhou , Y. Kong , H. Tan , Q. Huang , C. Wang , Z. Pei , H. Wang , Y. Liu , Y. Wang , S. Li , X. Liao , W. Yan , S. Zhao , Adv. Mater. 2022, 34, 2106541. 10.1002/adma.202106541 35191113

[26] a) B. Yan , D. Krishnamurthy , C. H. Hendon , S. Deshpande , Y. Surendranath , V. Viswanathan , Joule 2017, 1, 600;

[27] Z. Yang , C. Zhao , Y. Qu , H. Zhou , F. Zhou , J. Wang , Y. Wu , Y. Li , Adv. Mater. 2019, 31, 1808043. 10.1002/adma.201808043 30721541

[28] W. Ren , X. Tan , C. Jia , A. Krammer , Q. Sun , J. Qu , S. C. Smith , A. Schueler , X. Hu , C. Zhao , Angew. Chem., Int. Ed. Engl. 2022, 61, 202203335. 10.1002/anie.202203335 35315559

[29] J. Zhang , H. Yang , B. Liu , Adv. Energy Mater. 2021, 11, 2002473.

[30] a) Y.‐N. Gong , L. Jiao , Y. Qian , C.‐Y. Pan , L. Zheng , X. Cai , B. Liu , S.‐H. Yu , H.‐L. Jiang , Angew. Chem., Int. Ed. Engl. 2020, 59, 2705; 31821685 10.1002/anie.201914977

[31] a) C. Jia , X. Tan , Y. Zhao , W. Ren , Y. Li , Z. Su , S. C. Smith , C. Zhao , Angew. Chem., Int. Ed. Engl. 2021, 60, 23342; 34449125 10.1002/anie.202109373

[32] Y.‐Y. Ma , Z.‐L. Lang , L.‐K. Yan , Y.‐H. Wang , H.‐Q. Tan , K. Feng , Y.‐J. Xia , J. Zhong , Y. Liu , Z.‐H. Kang , Y.‐G. Li , Energy Environ. Sci. 2018, 11, 2114.

[33] Z. Zhang , J. Zhu , S. Chen , W. Sun , D. Wang , Angew. Chem., Int. Ed. Engl. 2023, 62, 202215136. 10.1002/anie.202215136 36399049

[34] Y. Mun , S. Lee , K. Kim , S. Kim , S. Lee , J. W. Han , J. Lee , J. Am. Chem. Soc. 2019, 141, 6254. 30920818 10.1021/jacs.8b13543

[35] X. Wang , X. Zhou , C. Li , H. Yao , C. Zhang , J. Zhou , R. Xu , L. Chu , H. Wang , M. Gu , H. Jiang , M. Huang , Adv. Mater. 2022, 34, 2204021. 10.1002/adma.202204021 35790038

[36] a) J. Wang , H. Li , S. Liu , Y. Hu , J. Zhang , M. Xia , Y. Hou , J. Tse , J. Zhang , Y. Zhao , Angew. Chem., Int. Ed. Engl. 2021, 60, 181; 32935443 10.1002/anie.202009991

[37] F. Wang , R. Zhang , Y. Zhang , Y. Li , J. Zhang , W. Yuan , H. Liu , F. Wang , H. L. Xin , Adv. Funct. Mater. 2023, 33, 2213863.

[38] C. H. Choi , M. Kim , H. C. Kwon , S. J. Cho , S. Yun , H. T. Kim , K. J. Mayrhofer , H. Kim , M. Choi , Nat. Commun. 2016, 7, 10922. 26952517 10.1038/ncomms10922 PMC4786782

[39] Y. J. Sa , J. H. Kim , S. H. Joo , Angew. Chem., Int. Ed. Engl. 2019, 58, 1100. 30548090 10.1002/anie.201812435

[40] a) Q. Tian , L. Jing , H. Du , Y. Yin , X. Cheng , J. Xu , J. Chen , Z. Liu , J. Wan , J. Liu , J. Yang , Nat. Commun. 2024, 15, 983; 38302469 10.1038/s41467-024-45243-w PMC10834542

[41] Y. Tian , M. Li , Z. Wu , Q. Sun , D. Yuan , B. Johannessen , L. Xu , Y. Wang , Y. Dou , H. Zhao , S. Zhang , Angew. Chem., Int. Ed. Engl. 2022, 61, 202213296. 10.1002/anie.202213296 PMC10098864 36280592

[42] S. Liu , Y. Zhang , B. Ge , F. Zheng , N. Zhang , M. Zuo , Y. Yang , Q. Chen , Adv. Mater. 2021, 33, 2103133. 10.1002/adma.202103133 34467573

[43] L. Kong , M. Wang , Y. Tuo , S. Zhou , J. Wang , G. Liu , X. Cui , J. Wang , L. Jiang , J. Energy Chem. 2024, 88, 183.

[44] a) X. Cheng , J. Yang , W. Yan , Y. Han , X. Qu , S. Yin , C. Chen , R. Ji , Y. Li , G. Li , G. Li , Y. Jiang , S. Sun , Energy Environ. Sci. 2021, 14, 5958;

[45] a) R. Lin , L. Kang , K. Lisowska , W. He , S. Zhao , S. Hayama , G. J. Hutchings , D. J. L. Brett , F. Corà , I. P. Parkin , G. He , Angew. Chem., Int. Ed. Engl. 2023, 62, 202301433; 10.1002/anie.202301433 PMC10962607 36947446

[46] J. Hu , W. Shang , C. Xin , J. Guo , X. Cheng , S. Zhang , S. Song , W. Liu , F. Ju , J. Hou , Y. Shi , Angew. Chem., Int. Ed. Engl. 2023, 62, 202304754. 10.1002/anie.202304754 37126395

[47] a) C. Tang , L. Chen , H. Li , L. Li , Y. Jiao , Y. Zheng , H. Xu , K. Davey , S.‐Z. Qiao , J. Am. Chem. Soc. 2021, 143, 7819; 33983725 10.1021/jacs.1c03135

