# Supplementary material for: Coupling Ni Single Atomic Sites with Metallic Aggregates at Adjacent Geometry on Carbon Support for Efficient Hydrogen Peroxide Electrosynthesis
Source: Adv Sci (Weinh). 2024 Apr 11;11(25):2402240. doi: 10.1002/advs.202402240 (PMC11220688; doi:10.1002/advs.202402240)
Supplement: Supplementary file 1 — Supporting Information [file ADVS-11-2402240-s001.pdf]

ADVANCED SCIENCE
Open Access

Supporting Information

for *Adv. Sci.*, DOI 10.1002/advs.202402240

Coupling Ni Single Atomic Sites with Metallic Aggregates at Adjacent Geometry on Carbon Support for Efficient Hydrogen Peroxide Electrosynthesis

Xin Wang, Run Huang, Xin Mao, Tian Liu, Panjie Guo, Hai Sun, Zhelin Mao, Chao Han, Yarong Zheng, Aijun Du, Jianwei Liu*, Yi Jia* and Lei Wang**

Supporting Information

Coupling Ni Single Atomic Sites with Metallic Aggregates at Adjacent Geometry on Carbon Support for Efficient Hydrogen Peroxide Electrosynthesis

Xin Wang, Run Huang, Xin Mao, Tian Liu, Panjie Guo, Hai Sun, Zhelin Mao, Chao Han, Yarong Zheng, Aijun Du, Jianwei Liu,* Yi Jia,* and Lei Wang**

X. Wang, R. Huang, P. Guo, H. Sun, Z. Mao, C. Han, Lei Wang

College of Chemical Engineering

Zhejiang University of Technology

Hangzhou 310014, P. R. China

E-mail: wangxin136@zjut.edu.cn, sumin224@zjut.edu.cn

X. Mao, A. Du

School of Chemistry, Physics and Mechanical Engineering

Queensland University of Technology

Brisbane, QLD 4000, Australia

T. Liu, J. Liu

Division of Nanomaterials & Chemistry, Hefei National Research Center for Physical Sciences at the Microscale, Institute of Energy, Hefei Comprehensive National Science Center, Department of Chemistry, Institute of Biomimetic Materials & Chemistry, Anhui Engineering Laboratory of Biomimetic Materials

University of Science and Technology of China

Hefei, 230026 China

E-mail: jwliu13@ustc.edu.cn

Y. Zheng

Anhui Province Key Laboratory of Advanced Catalytic Materials and Reaction Engineering, School of Chemistry and Chemical Engineering

Hefei University of Technology

Hefei, China

Y. Jia

Petroleum and Chemical Industry Key Laboratory of Organic Electrochemical Synthesis, College of Chemical Engineering

Zhejiang Carbon Neutral Innovation Institute

Zhejiang University of Technology (ZJUT)

Hangzhou 310014, P. R. China

Moganshan Institute ZJUT

Deqing 313200, P. R. China

Email: jiayi@zjut.edu.cn

1. Chemicals.

All reagents were obtained from commercial sources and used as received without further purification. Melamine ($C_3H_6N_6$, 99%) was purchased from Aladdin. Potassium hydroxide (KOH, ≥85%), anhydrous ethanol (C_2H_5OH, HPLC) and hydrochloric acid (HCl, 37%) were purchased from Titan Technology. Hexamine ($C_6H_{12}N_4$, ≥98%),dibenzyl disulfide ($C_{14}H_{14}S_2$, 98%) and nickel nitrate hexahydrate ($Ni(NO_3)_2 \cdot 6H_2O$, ≥98%) were obtained from Sinopharm Chemical. Boric acid (H_3BO_3, ≥99.5%) was purchased from Maclin Biochemical Technology. Sodium dihydrogen phosphate (NaH_2PO_4, ≥98%) and potassium thiocyanate (KSCN, 99.5%) were obtained from Merrier Biochemical Technology. Nafion (5 wt%) was obtained from Alfa Essa.

2. Material synthesis

Preparation of NiO nanosheet.

1.0 g of $Ni(NO_3)_2 \cdot 6H_2O$ and 1.0 g of hexamine (HMT) were first dissolved in 20 mL of ultrapure water to form a clear solution, which was then transferred into a Teflon-lined autoclave with volume of 50 mL and heated at 95 °C for 9 h. After carefully washing and drying, the obtained powder was placed in a porcelain boat and heated at 350 °C for 2 h in a muffle furnace to generate the NiO nanosheet.

Preparation of C_3N_4.

6 g of melamine was placed in a porcelain boat and then heated at 550 °C for 2 h in a muffle furnace to obtain C_3N_4 (a yellow block).

Preparation of Ni_{SA}/Ni_{NP}-NCNT.

The mixture powder of the as-synthesized NiO and C_3N_4 at a mass ratio of 1:100 was loaded into a tubular furnace. Subsequently, the mixture was heated from room temperature to 900 °C at a rate of 2 °C min^{-1} under a N_2 atmosphere. Following this, the mixture was kept at 900 °C for 5 minutes before being cooled back to room temperature. The resulting material was identified as Ni_{SA}/Ni_{NP}-NCNT. Similarly, using the same procedure, control samples were prepared at different temperatures while keeping the other parameters constant, except for the temperature variation, which were denoted as Ni_{SA}/Ni_{NP}-NCNT-800 and Ni_{SA}/Ni_{NP}-NCNT-1000, respectively.

Preparation of Ni_{SA}/Ni_{NP}-NSCNT, Ni_{SA}/Ni_{NP}-NPCNT and Ni_{SA}/Ni_{NP}-NBCNT.

The above Ni_{SA}/Ni_{NP}-NCNT was mixed with dibenzyl disulfide in a certain mass ratio (mass ratio 1:1) and placed in a tube furnace. Under the protection of N_2 atmosphere, the mixture was heated from room temperature to 350 °C at a rate of 2°C min^{-1}, held at this temperature for 120 min, and then cooled to room temperature. The resulting black powder was then treated with 3 M HCl for 24 h to remove exposed metal aggregations. After washing with deionized water for three times, the Ni_{SA}/Ni_{NP}-NSCNT sample was obtained. The Ni_{SA}/Ni_{NP}-NPCNT and Ni_{SA}/Ni_{NP}-NBCNT samples can be synthesized via a similar procedure as mentioned above, except using sodium hypophosphite and boric acid as dopants, respectively. Notablly, the heating temperature was set to 350 and 600 °C for the production of Ni_{SA}/Ni_{NP}-NPCNT and Ni_{SA}/Ni_{NP}-NBCNT, respectively. By using Ni_{SA}/Ni_{NP}-NCNT-800 and Ni_{SA}/Ni_{NP}-NCNT-1000 as precursors, Ni_{SA}/Ni_{NP}-NXCNT-800, Ni_{SA}/Ni_{NP} -NXCNT-1000 (X refers to the P or B element) can also be prepared.

Preparation of R-Ni_{SA}/Ni_{NP}-NSCNT, R-Ni_{SA}/Ni_{NP}-NPCNT and R-Ni_{SA}/Ni_{NP}-NBCNT

The Ni_{SA}/Ni_{NP}-NCNT was first treated with 3M HCl to remove the exposed Ni nanoparticles. Then, doping correspoonding heteroatoms into carbon matrix following the procedure as mentioned above could produce the R-Ni_{SA}/Ni_{NP}-NSCNT, R-Ni_{SA}/Ni_{NP}-NPCNT and R-Ni_{SA}/Ni_{NP}-NBCNT.

Preparation of Ni-NSC

Ni-NSC catalysts were prepared according to the reported literature[1]. Solution A, comprising 491.0 mg of $Zn(NO_3)_2 \cdot 6H_2O$ and 276.0 mg of dibenzothiophene dissolved in 35.0 mL of MeOH, was prepared, while Solution B, involving 620.0 mg of 2-methylimidazole dissolved in 10.0 mL of MeOH, was separately formulated. Upon creation of these solutions, Solution B was meticulously added dropwise to solution a under continuous stirring over a period of 1 hour. Subsequently, the resultant S-doped ZIF-8 was retrieved through centrifugation, subjected to thorough washing, and thereafter dried and set aside for further processing. The acquired S-doped ZIF-8 was subsequently subjected to a heating process in a tube furnace at 1000°C for 2 hours, with a heating rate of 5 °C min^{-1}, under a N_2 atmosphere to yield NSC. Following this, 100.0 mg of NSC was immersed in a solution comprising 86.7 mL of isopropanol and reverse osmosis (RO) water at a 1:1 volume ratio. Moreover, a new solution, designated as Solution C, was prepared by blending 200.0 mL of aqueous isopropanol/RO mixture (with a 1:1 volume ratio) containing 124.4 mg of $Ni(OAc)_2 \cdot$4H2O and 290.0 mg of 1,10-phenanthroline. Subsequently, Solution C (13.35 mL) was introduced into the

previously prepared dispersion, followed by stirring at room temperature for a period of 6 hours. The solvent was subsequently eliminated using a rotary evaporator and the residues were dried under vacuum at 60 °C. The resulting powder was then subjected to calcination under an Ar atmosphere at 800 °C for 2 hours, ramping up to temperature at a rate of 5 °C min^{-1}. The final product derived from this intricate process was termed Ni-NSC.

3. Characterization

Scanning electron microscope (SEM) images were obtained on a Zeiss Geminisem 500 microscope at 5 kV. Transmission electron microscopy (TEM), high resolution TEM (HR-TEM), high angle annular dark field scanning transmission electron microscopy and energy dispersive X-ray spectroscopy (EDX) mapping images were obtained on the JEOL JEM-2100F 300kV. X-ray diffraction (XRD) patterns were recorded using Rigaku Ultima IV operating at 40 kV with Cu Kα radiation (λ = 0.15406 nm), a theta range of 5° to 90° and a scan rate of 10°/min. Raman spectra were collected on a Raman spectrometer system (Raman spectrometer model: HORIBA HR Evolution) using a laser with a wavelength of 532nm. Brunauer-Emmett-Teller (BET) surface area of the obtained material was measured using a relative pressure of P/P_0 = 0.05-0.1. X-ray photoelectron spectroscopy (XPS) was carried out on a Thermo Scientific K-Alpha instrument, which operates at 12 kV using Al-Ka radiation. The Ni-K-edge XANES spectra of the catalysts were measured at the optical emission end station of BSRF's beamline 4B9A in Beijing, China.

4. Electrochemical measurements

The electrochemical tests are all carried out using a three-electrode system via the CHI760E electrochemical workstation. The rotating ring disc electrode (RRDE) measurement consists of a rotating disc electrode (glassy carbon disc, 4 mm diameter), a rotating ring disc (glassy carbon disc, 5 mm inner diameter, 7 mm outer diameter, Pt ring), a platinum wire and an Ag/AgCl electrode (saturated KCl solution) as working electrode. The platinum wire was used as the counter electrode and the Ag/AgCl electrode as the reference electrode. The reference electrode Ag/AgCl (saturated KCl solution) was calibrated relative to the reversible hydrogen electrode (RHE), $E_{RHE} = E_{Ag/AgCl} + 0.960$. A catalyst ink was made by sonicating 5 mg of sample, 10 ul of Nafion (5 wt %), 700 ul of water and 290 ul of isopropanol for 1 h. Afterwards, 5 ul of the dispersion was dropped onto the disc electrode and then dried at room temperature. The mass loading of the catalyst at the disc electrode was 0.20 mg cm^{-2}. O_2 was passed into 0.1 M KOH for 30 min before testing to ensure O_2 saturation was achieved. Linear scanning voltammetry (LSV) tests were performed at 1600 rpm in an O_2 saturated 0.1M KOH electrolyte. Repeat at a scan rate of 10mV s^{-1} over a potential range of 0.1V vs. RHE to 1.0V vs. RHE, The ring potential was also held at 1.2V vs.RHE. Normalise all current densities to the area of the disc electrode.

In performing Ni single atom masking experiments to verify the active site test. Change the electrolyte from 0.1 M KOH to 0.5 ml 1 M KSCN + 0.1 M KOH. The rest of the procedure is the same.

The hydrogen peroxide yield (H_2O_2 (%)) and the number of electron transfers (n) are calculated based on the following equations:

$$n = 4 \times \frac{I_d}{I_{d+I_r/N}}$$

$$H_2O_2(\%) = 200 \times \frac{I_r/\mathrm{N}}{I_d + I_r/\mathrm{N}}$$

where I_d is the disk current, I_r is the ring current and N = 0.42 is the current collection efficiency of the platinum ring.

Electrochemical test in the H-cell. Experiments on H_2O_2 production were carried out in an H-cell electrolyser using a three-electrode structure:Ni_{SA}/Ni_{NP}-NSCNT electrocatalyst-coated carbon paper, carbon rods, and Ag/AgCl electrodes as the working electrode, counting electrode, and reference electrode, respectively. The contact area of the carbon paper working electrode with the electrolyte was 1 cm^2. Nafion 117 anion exchange membrane was used to separate the cathode and anode chambers. The H_2O_2 yield was determined using the colorimetric method:$2Ce^{4+} + H_2O_2 \rightarrow 2Ce^{3+} + 2H^+ + O_2$ and cerium sulfate $Ce(SO_4)_2$ titration. A standard UV absorption curve was calibrated with a series of $Ce(SO_4)_2$ solutions of known concentration (0.1, 0.15, 0.2, 0.3 and 0.4mM).The yield of H_2O_2 can be calculated using the following equation:

$$\mathrm{H_2O_2\ productivity} = \frac{\mathrm{CV}}{\mathrm{mh}}$$

$$\mathrm{C} = \frac{\mathrm{C_1V_1 - C_2V_2}}{\mathrm{2V_3}}$$

where C is the concentration of H_2O_2 in the electrolyte, V is the total volume of electrolyte in the cathode chamber, m is the catalyst loading, and h is the test time. At the end of the test, 0.5 mL of electrolyte was titrated into 5 mL of $Ce(SO_4)_2$ solution and 4.5 mL of sulfuric acid solution.In the experiment, V in the cathode half-cell was

65 mL, and t was set to 1h. The cathode half-cell was used in the experiment. C_1 is the concentration of Ce^{4+} before the reaction, V_1 is the volume of Ce^{4+} before the addition of the reaction; C_2 is the concentration of Ce^{4+} remaining after the reaction, V_2 is the total volume of the electrolyte after the addition of the electrolyte. V_3 is the volume of the electrolyte added.

5. Computational details

Density functional theory as implemented in the Vienna Ab-initio Simulation Package (VASP) was employed to optimize geometry structures[2]. The exchange-correlation interactions were described by the generalised gradient approximation (GGA)[3] in the form of the Perdew-Burke-Ernzerhof functional (PBE)[4]. A cut-off energy of 500 eV for plain-wave basis sets was adopted and the convergence threshold was 10^{-5} eV, and 5×10^{-3} eV/Å for energy and force, respectively. The weak interaction was described by DFT+D3 method using empirical correction in Grimme′s scheme.[5] The vacuum space was set to be more than 20 Å, which was enough to avoid the interaction between periodical images.

The reaction Gibbs free energy changes (ΔG) for each elementary steps were based on the computational hydrogen electrode model, which can be calculated by the following equation;

$$\Delta G = \Delta E + \Delta ZPE - T\Delta S$$

where ΔG is obtained directly from DFT calculations, ΔZPE is the change of zero-point energies (ZPE), T is the temperature of 298.15K, and ΔS is the change in entropy of products and reactants.

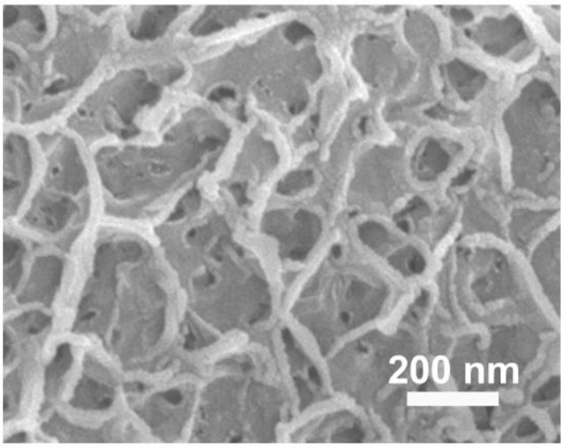

Figure S1. SEM image of C_3N_4.

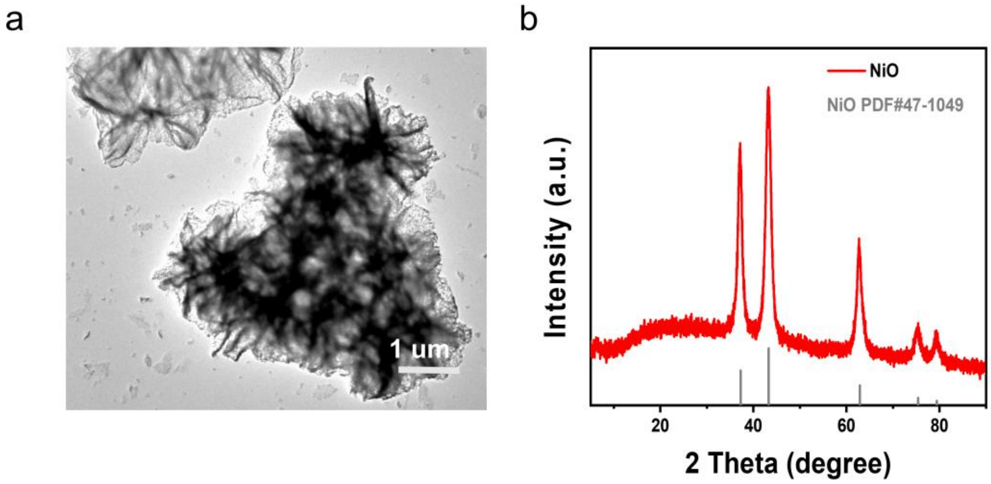

Figure S2. SEM image a) and XRD pattern b) of as-prepared NiO nanosheets.

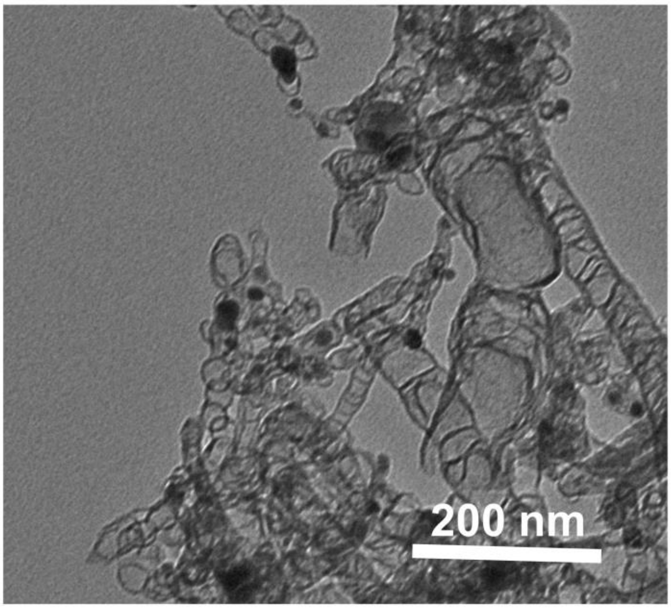

Figure S3. TEM image of Ni_{SA}/Ni_{NP}-NSCNT, indicating that small nanoparticles are distributed randomly at the tips or inside within carbon nanotubes.

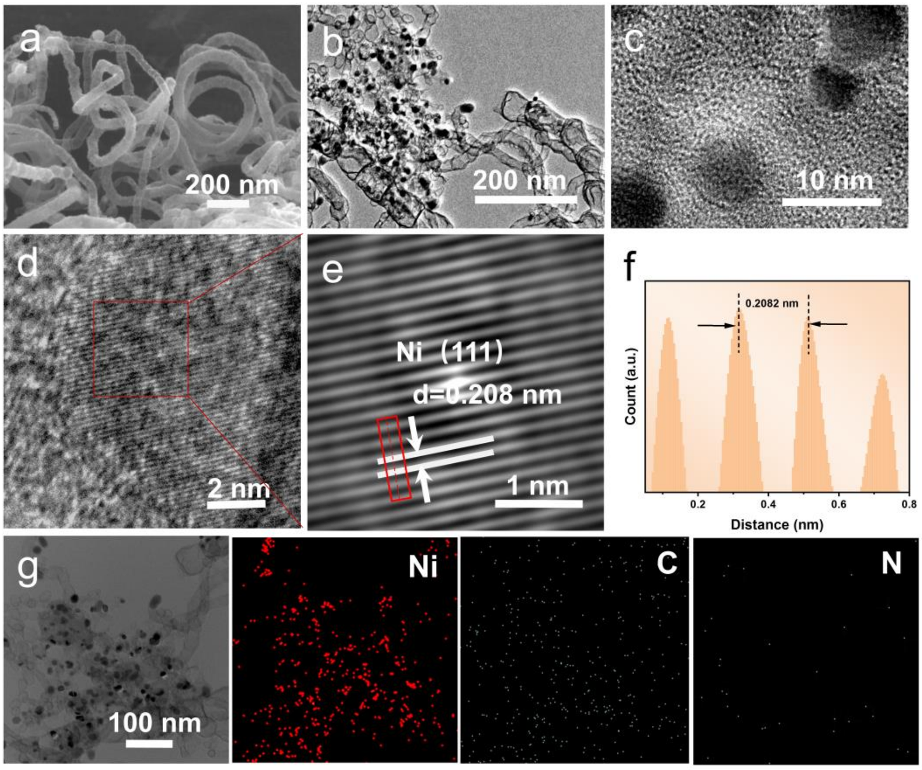

Figure S4. Morphological structure of Ni_{SA}/Ni_{NP}-NCNT catalysts. a) SEM image of Ni_{SA}/Ni_{NP}-NCNT, b) and c) are TEM and HRTEM image of Ni_{SA}/Ni_{NP}-NCNT, d) HRTEM image and its e) corresponding IFFT images and f) lattice plane spacing (All due to enlargement in the red dotted box), g) Elemental mapping of Ni_{SA}/Ni_{NP} -NCNT. Ni (blue), N (purple), C (red).

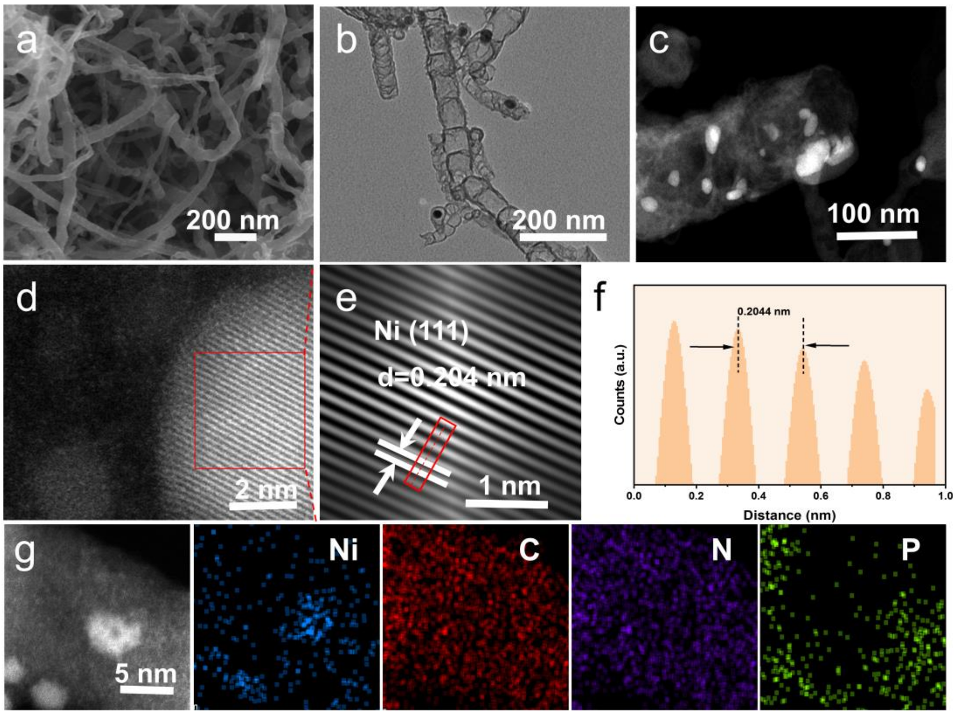

Figure S5. Morphological structure of Ni_{SA}/Ni_{NP}-NPCNT catalysts. a) SEM image of Ni_{SA}/Ni_{NP}-NPCNT. b) and c) are TEM and HAADF-STEM image of Ni_{SA}/Ni_{NP}-NPCNT. d) HRTEM image and e) corresponding IFFT images and f) lattice plane spacing (All due to enlargem ent in the red dotted box), g) Elemental mapping of Ni_{SA}/Ni_{NP}-NPCNT. Ni (blue), N (purple), P (green), C (red).

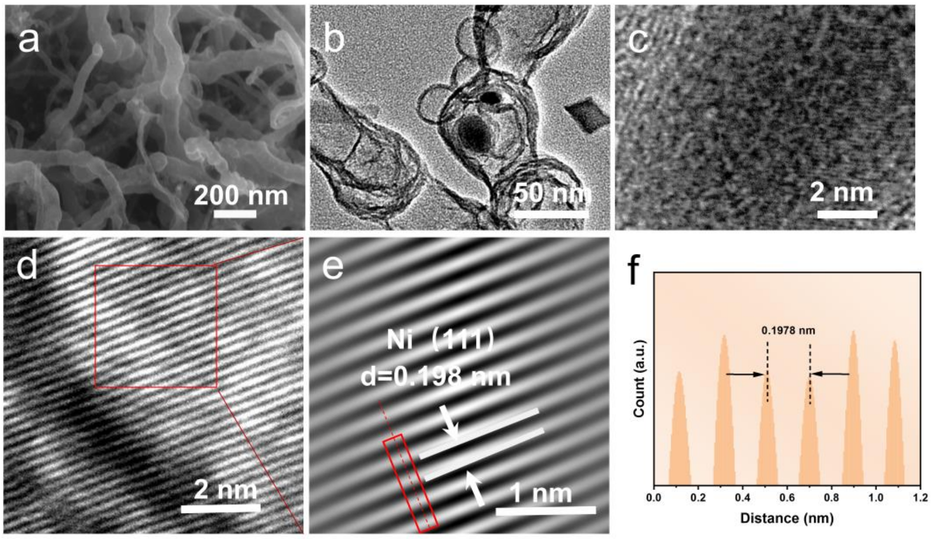

Figure S6. Morphological structure of Ni_{SA}/Ni_{NP}-NBCNT catalysts. a) SEM image of Ni_{SA}/Ni_{NP}-NBCNT, b) and c) are TEM and HRTEM image of Ni_{SA}/Ni_{NP}-NBCNT, d) HRTEM image and its e) corresponding IFFT images and f) lattice plane spacing (All due to enlargem ent in the red dotted box).

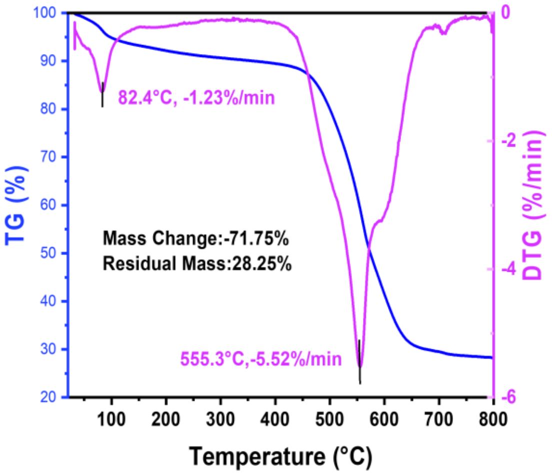

Figure S7. Thermogravimetric-differential scanning calorimetry (TG-DSC) analysis of C_3N_4/NiO mixture.

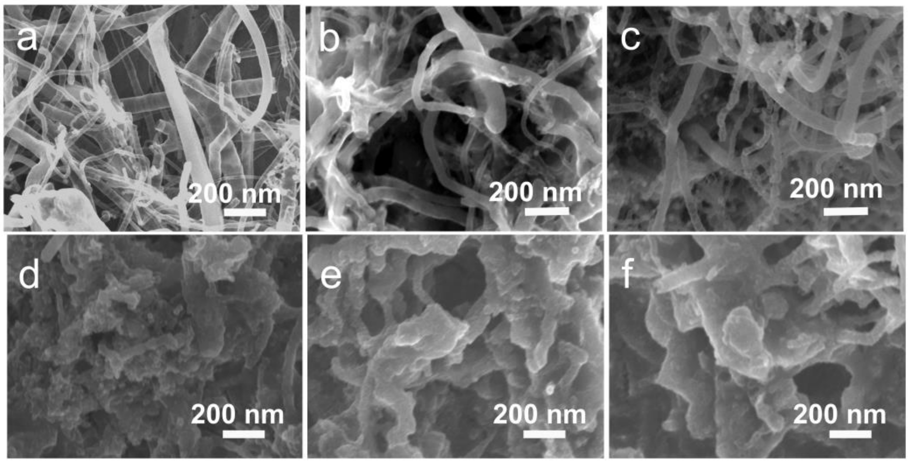

Figure S8. Morphological structure of samples prepared by pyrolysis at different temperatures. SEM image of a) Ni_{SA}/Ni_{NP}-NCNT-800, b) Ni_{SA}/Ni_{NP}-NPCNT-800, c) Ni_{SA}/Ni_{NP}-NS CNT-800 d) Ni_{SA}/Ni_{NP}-NC-1000, e) Ni_{SA}/Ni_{NP}-NPC-1000, f) Ni_{SA}/Ni_{NP}-NSC-1000.

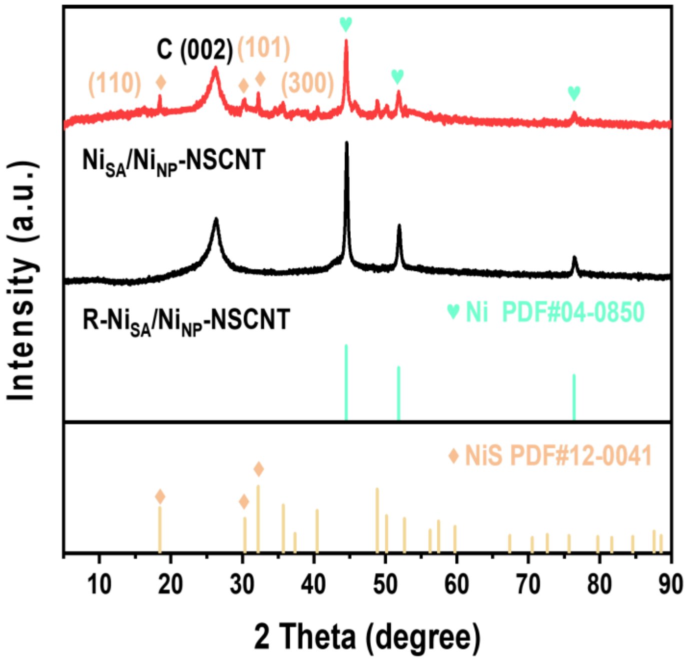

Figure S9. XRD pattern of the Ni_{SA}/Ni_{NP}-NSCNT and R-Ni_{SA}/Ni_{NP}-NSCNT.

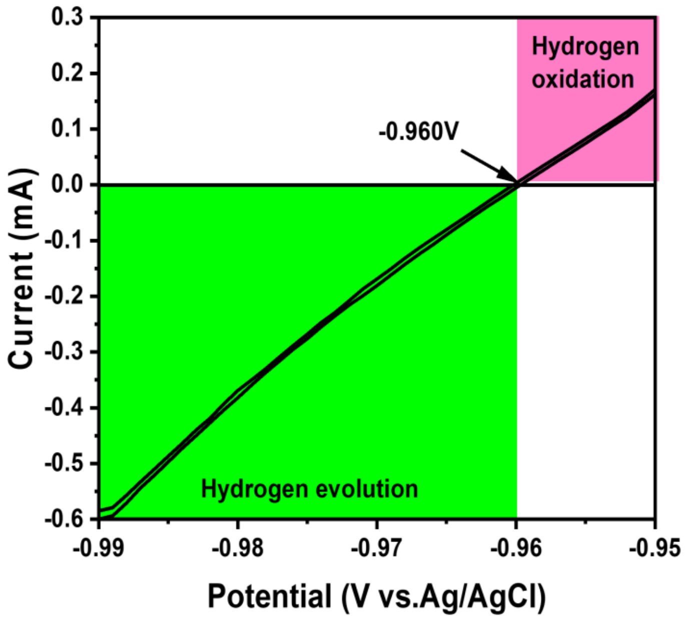

Figure S10. CV curve in 0.1 M KOH solutions under H_2-saturated condition with a Pt-foil as the working electrode for RHE calibration.

Based on the test, in 0.1 M KOH solutions, E(RHE) = E(Ag/AgCl) + 0.960 V.

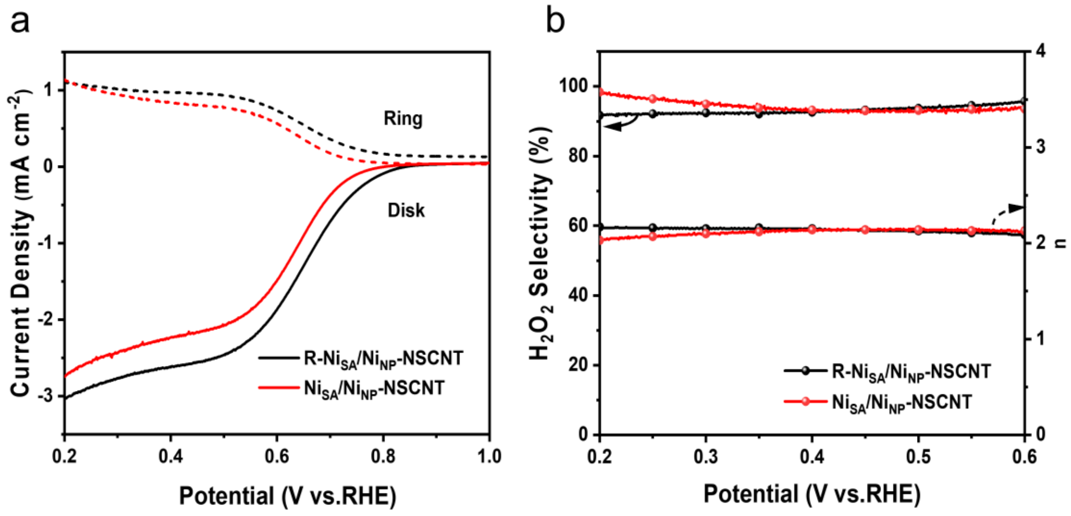

Figure S11. Comparison of ORR performance between Ni_{SA}/Ni_{NP}-NSCNT and R-Ni_{SA}/Ni_{NP}-NSCNT. a) Polarization curves. b) The calculated H_2O_2 selectivity and transfer electron numbers (n).

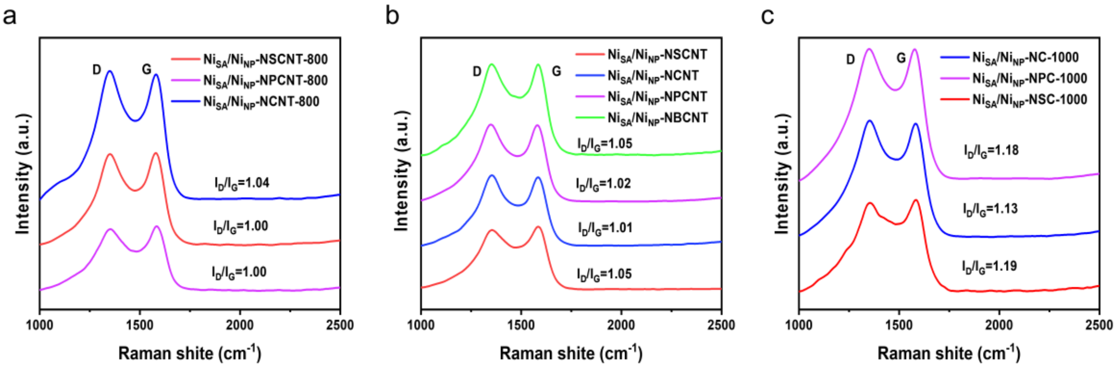

Figure S12. Raman spectra of as-made four samples (Ni_{SA}/Ni_{NP}-NCNT, Ni_{SA}/Ni_{NP}-NSCNT, Ni_{SA}/Ni_{NP}-NPCNT and Ni_{SA}/Ni_{NP}-NBCNT).

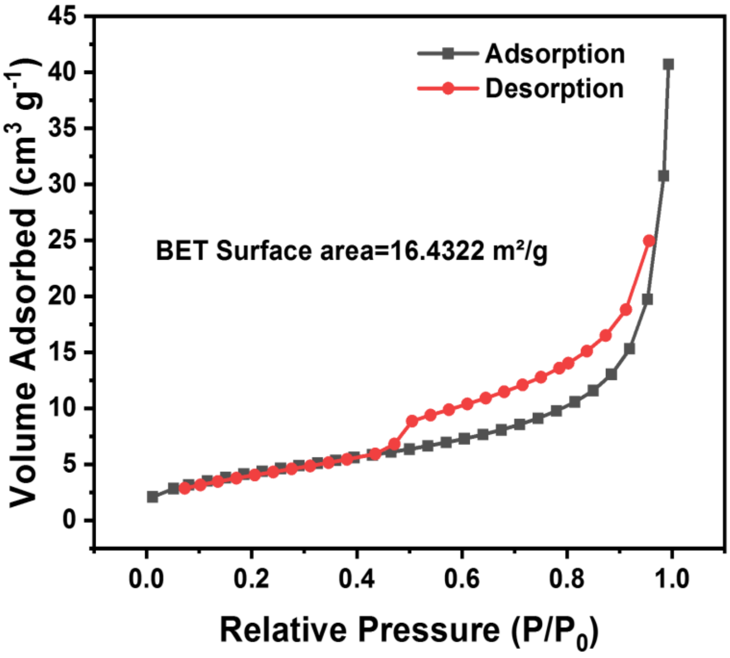

Figure S13. The N_2 adsorption/desorption curve of Ni_{SA}/Ni_{NP}-NSCNT.

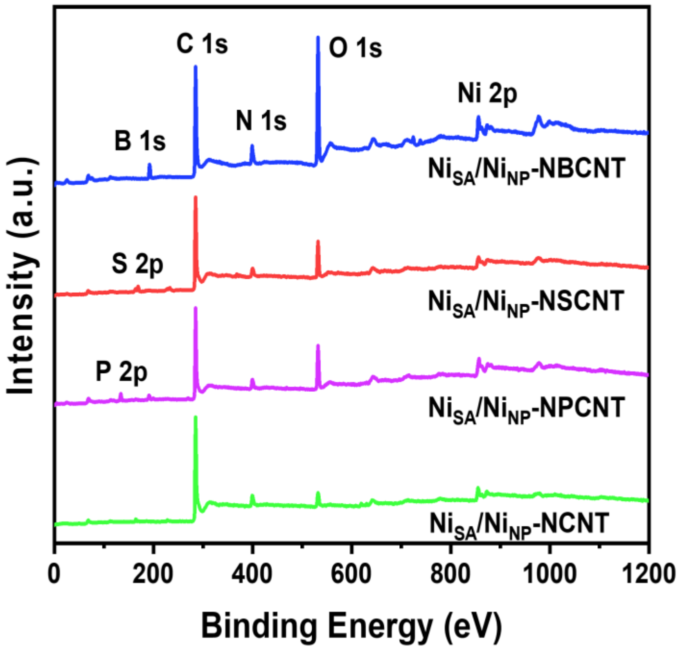

Figure S14. XPS survey spectra of as-prepared four catalysts.

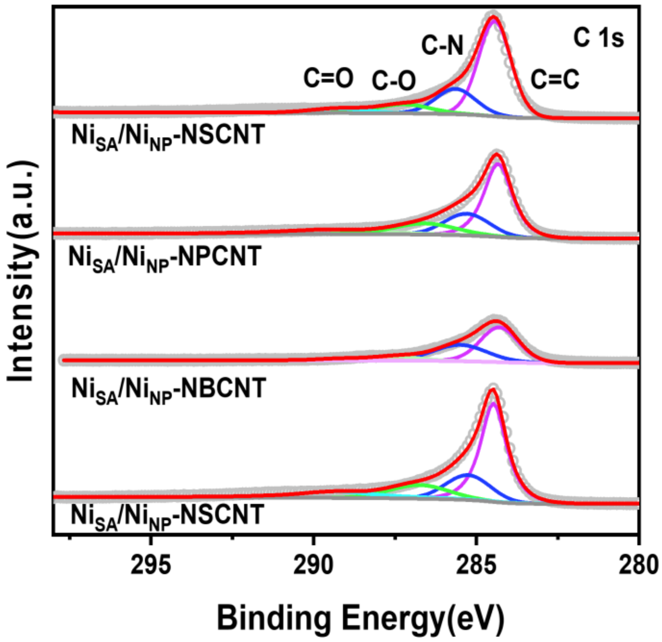

Figure S15. The C 1s XPS spectra of as-made four samples (Ni$_{SA}$/Ni$_{NP}$-NCNT, Ni$_{SA}$/Ni$_{NP}$-NSCNT, Ni$_{SA}$/Ni$_{NP}$-NPCNT and Ni$_{SA}$/Ni$_{NP}$-NBCNT).

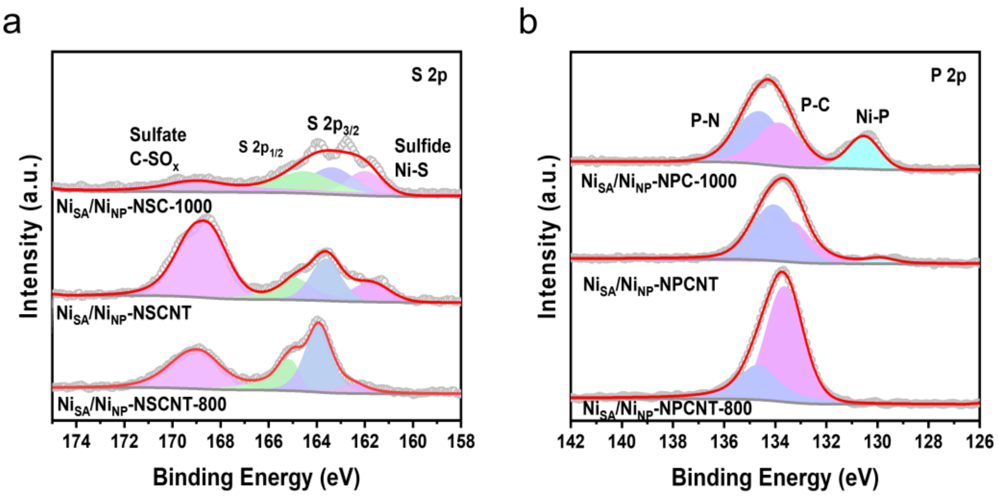

Figure S16. a) The S 2p XPS spectra of Ni_{SA}/Ni_{NP}-NSCNT samples prepared with different temperature (800-1000 °C) for the first-step thermal treatment. b) The P 2p XPS spectra of Ni_{SA}/Ni_{NP}-NPCNT samples prepared with different temperature (800-1000 °C) for the first-step thermal treatment.

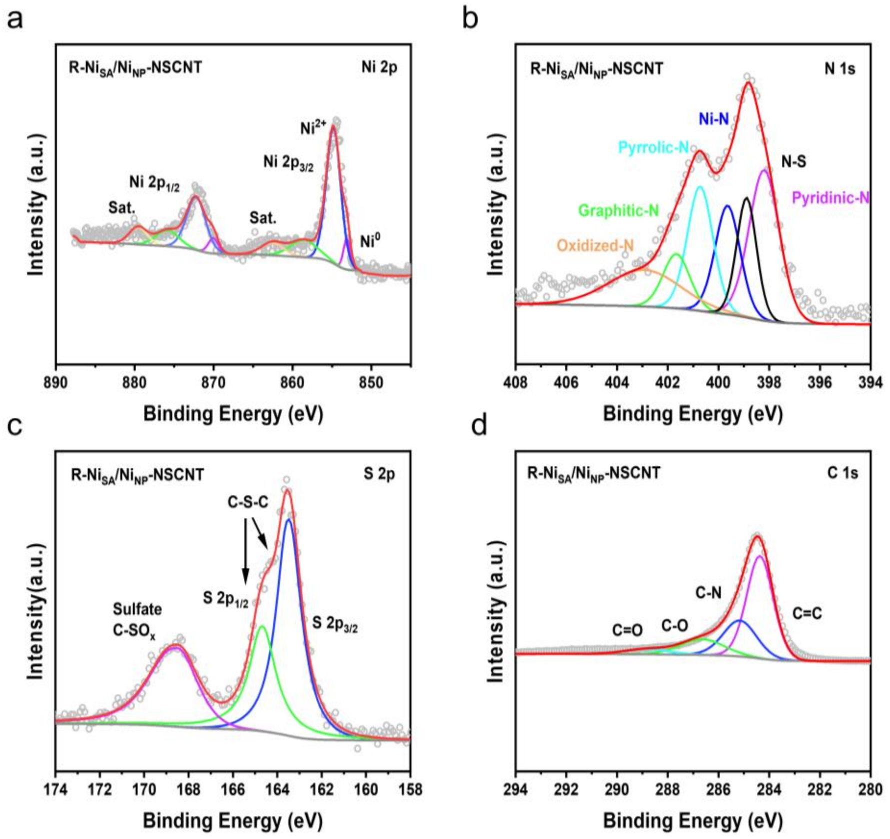

Figure S17. Deconvoluted XPS spectra of R-Ni_{SA}/Ni_{NP}-NSCNT for a) Ni 2p, b) N 1s, c) S 2p and d) C 1s.

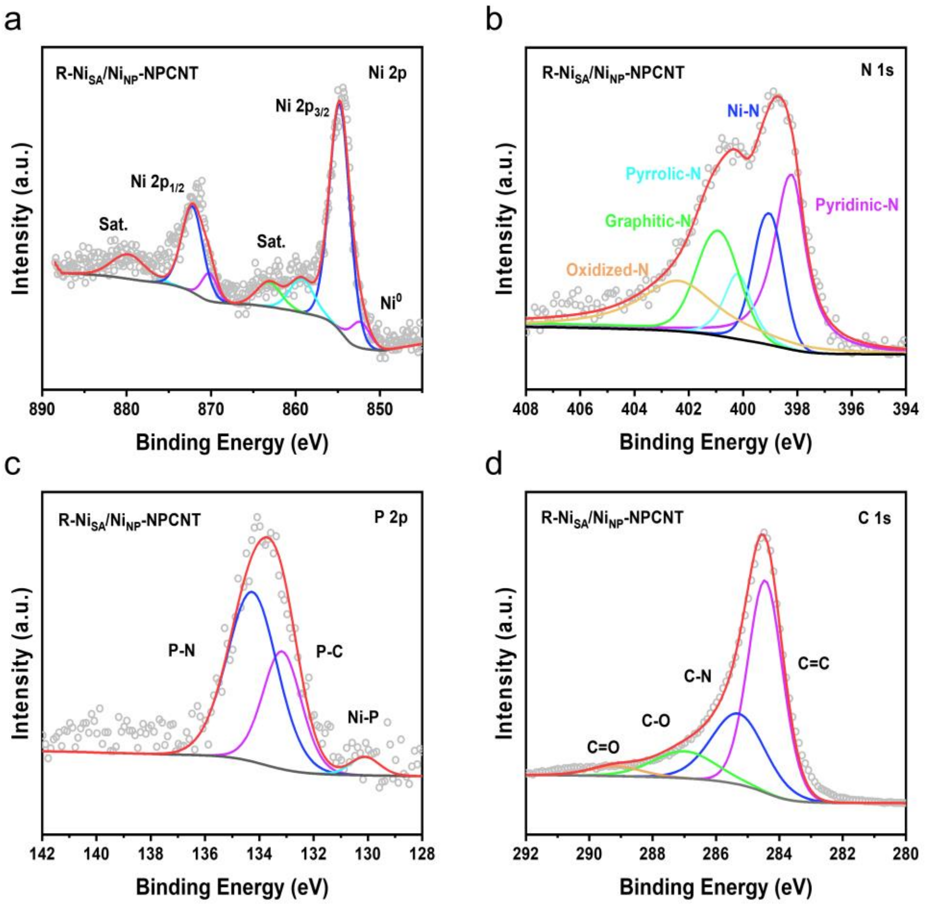

Figure S18. Deconvoluted XPS spectra of R-Ni$_{SA}$/Ni$_{NP}$-NPCNT for a) Ni 2p, b) N 1s, c) P 2p and d) C 1s.

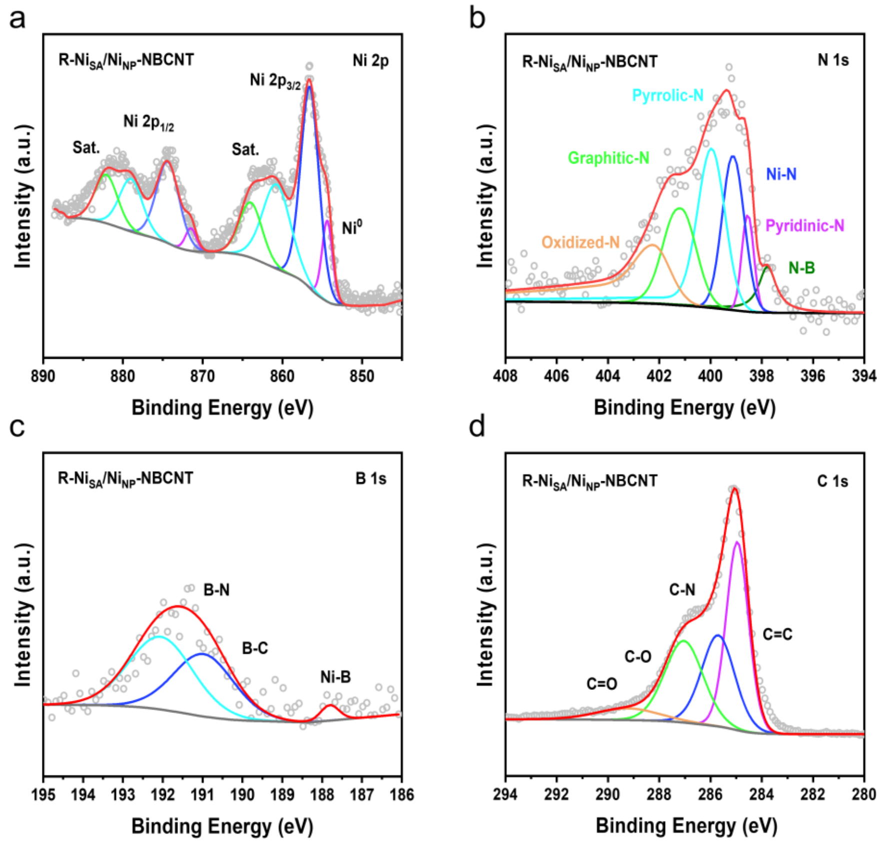

Figure S19. Deconvoluted XPS spectra of R-Ni$_{SA}$/Ni$_{NP}$-NBCNT for a) Ni 2p, b) N 1s, c) B 1s and d) C 1.

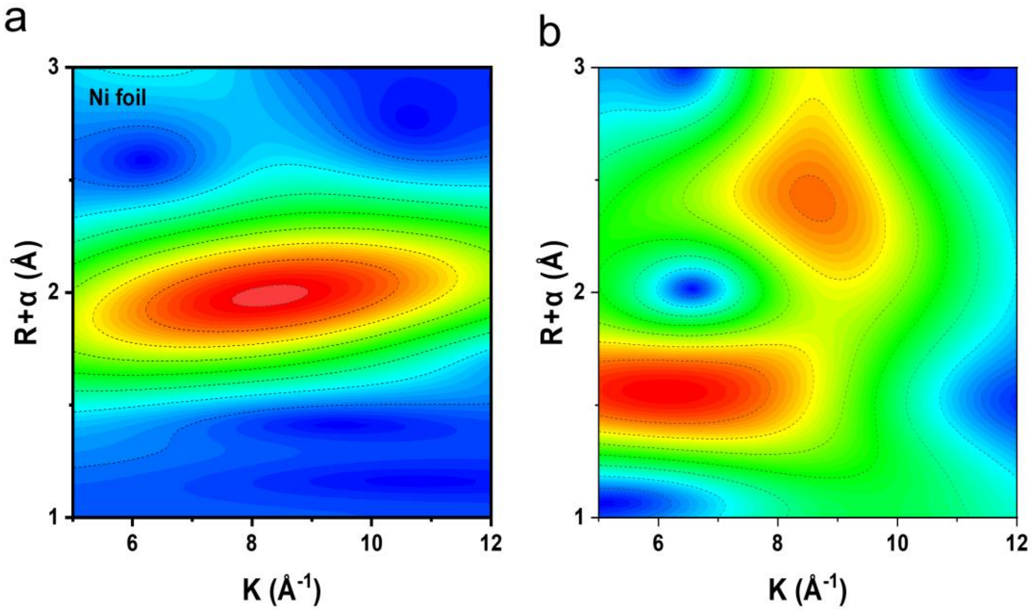

Figure S20. Wavelet transforms of the EXAFS spectra of Ni foil and NiPc.

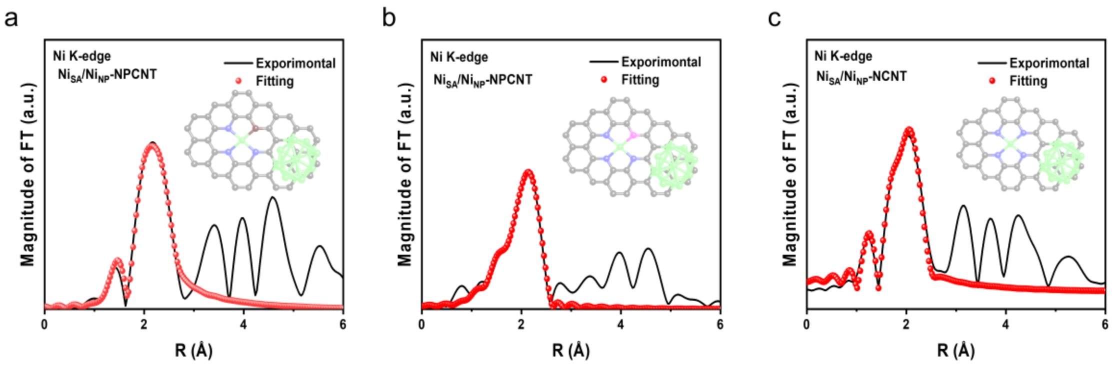

Figure S21. EXAFS fitting curves of a) Ni_{SA}/Ni_{NP}-NBCNT, b) Ni_{SA}/Ni_{NP}-NPCNT and c) Ni_{SA}/Ni_{NP}-NCNT.

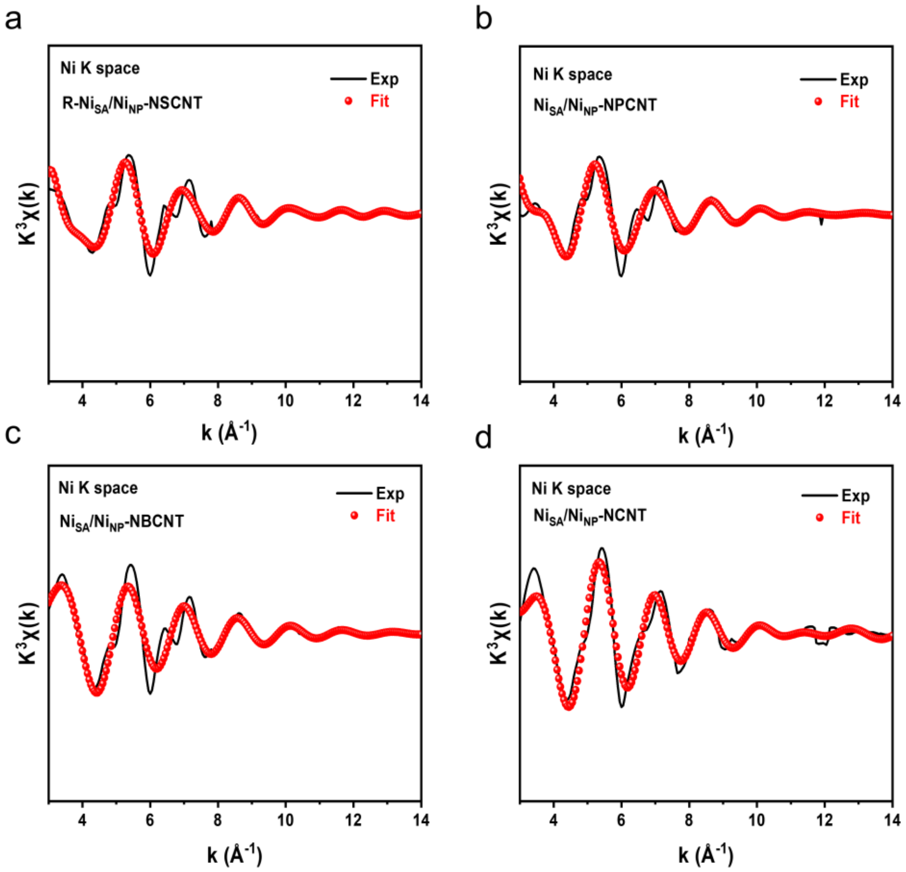

Figure S22. Ni K-space fitting curves of a) R-Ni$_{SA}$/Ni$_{NP}$-NSCNT b) Ni$_{SA}$/Ni$_{NP}$- NPCNT, c) Ni$_{SA}$/Ni$_{NP}$-NBCNT and d) Ni$_{SA}$/Ni$_{NP}$-NCNT.

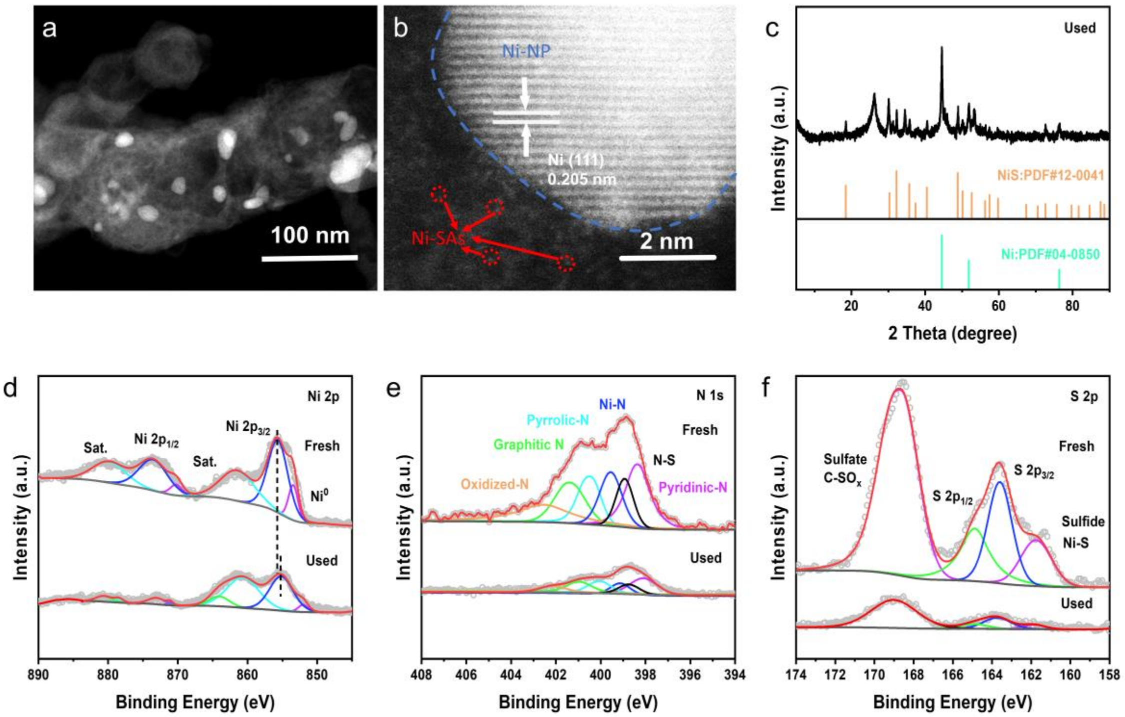

Figure S23. Characterization of Ni_{SA}/Ni_{NP}-NSCNT catalysts after ADT stability test. a, b) atomic-resolution HAADF-STEM images. c) XRD pattern. d-f) The high-resolution XPS spectra of Ni 2p, N 1s and S 2p.

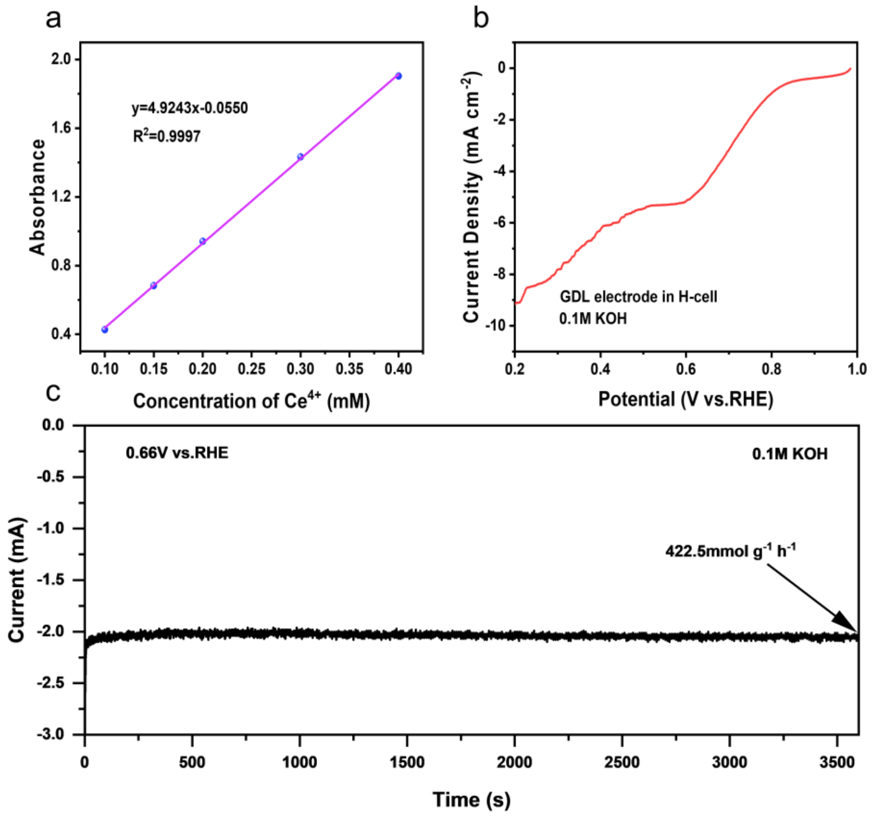

Figure S24. Bulk H_2O_2 production performance on Ni_{SA}/Ni_{NP}-NSCNT with a home-made H-cell in 0.1 M KOH electrolyte. a) UV-Vis spectrometer was used to determine different Ce^{4+} concentrations and adsorption at 320 nm was recorded. b) LSV polarization curve. c) Ampere i-t curves at 0.66V vs. RHE potential.

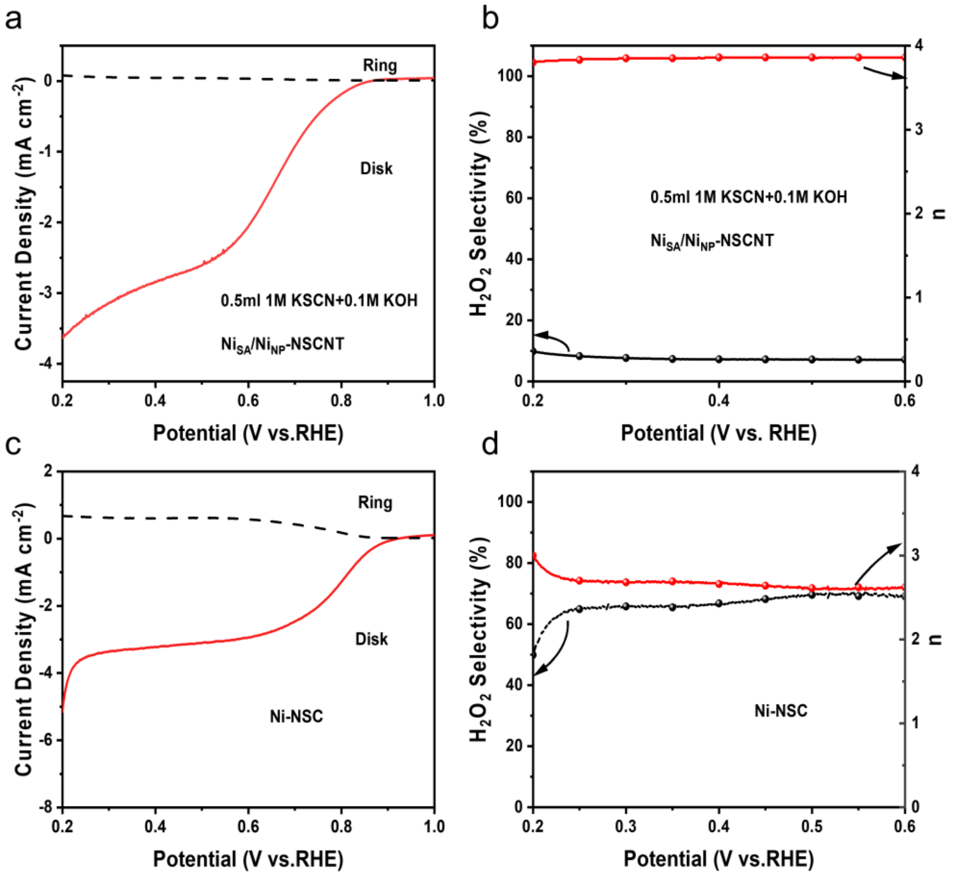

Figure S25. Evaluation of ORR performance for carbon-supported Ni nanoparticles and Ni single atoms. a) Polarization curves of Ni_{SA}/Ni_{NP}-NSCNT after adding KSCN to poison the single atomic sites. b) The calculated H_2O_2 selectivity and transfer electron number (n). c) Polarization curves of Ni-NSC.d) The calculated H_2O_2 selectivity and transfer electron number (n).

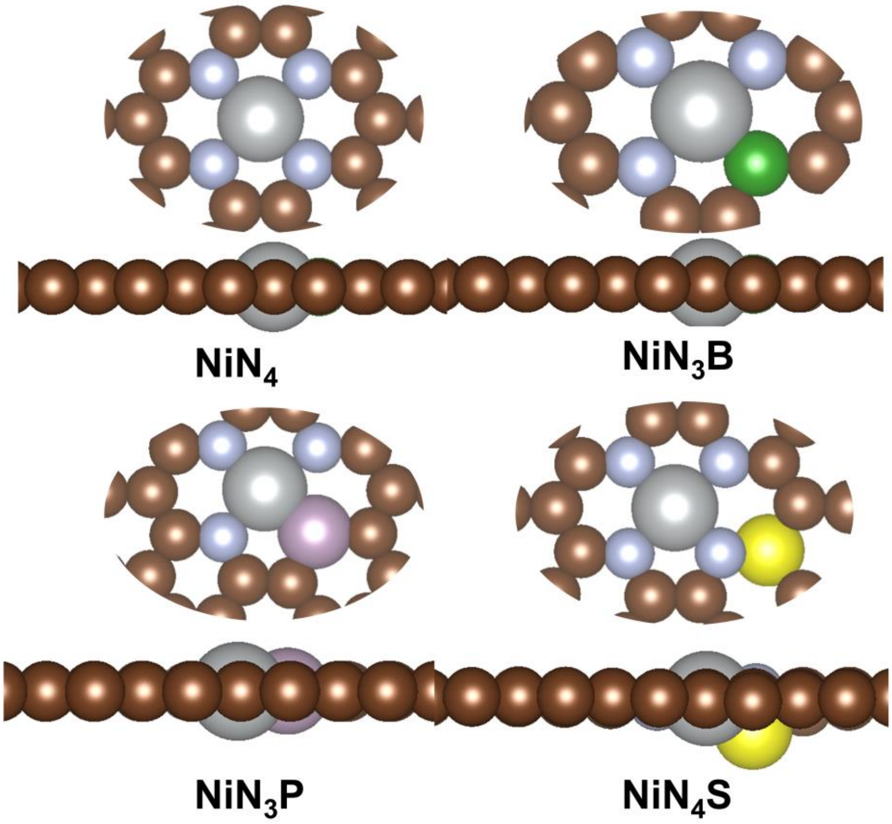

Figure S26. The constructed model structures with only single Ni atomic sites, referred as NiN_4, NiN_3B, NiN_3P and NiN_4S. Brown, gray, yellow, green, blue and purple spheres represent C, Ni, S, B, N and P atoms, respectively.

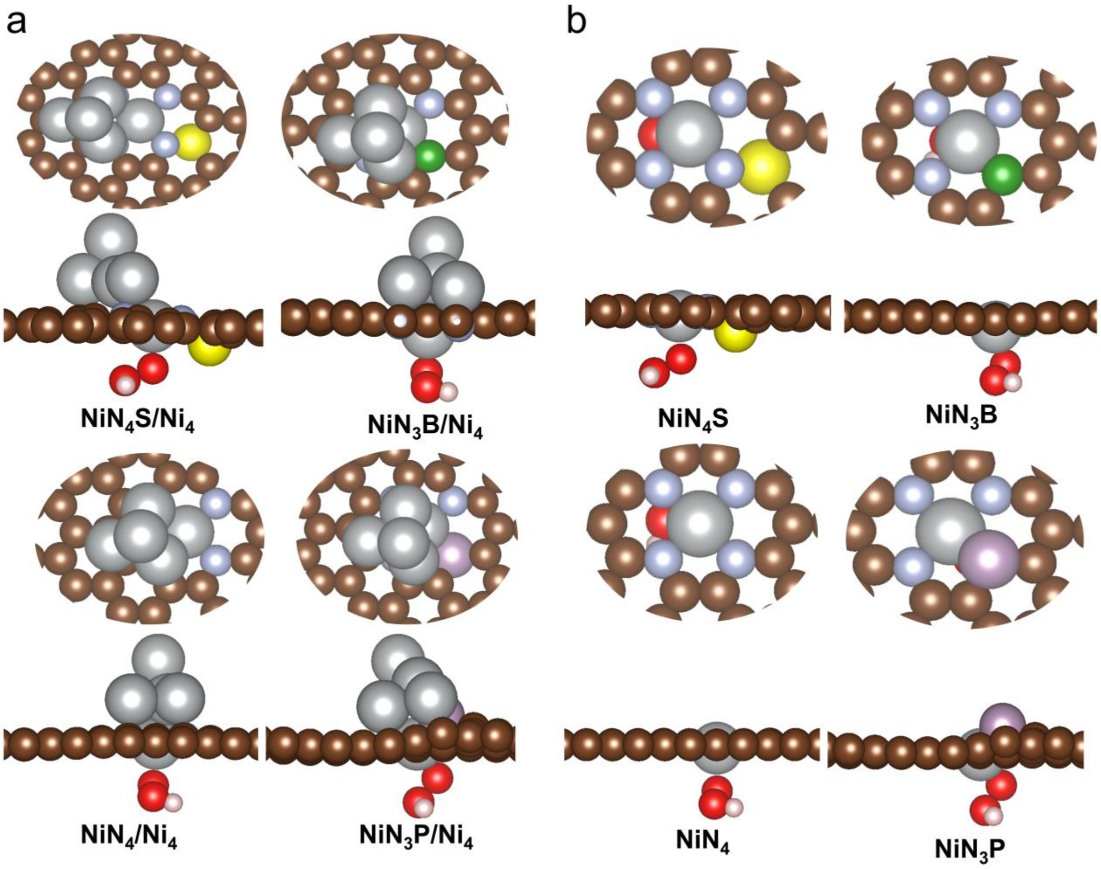

Figure S27. Top and side views of *OOH adsorption configurations on each model NiN_4S/Ni_4, NiN_3B/Ni_4, NiN_4/Ni_4, NiN_3P/Ni_4, NiN_4S, NiN_3B, NiN_4 and NiN_3P, where red represents O atoms, gray represents Ni atoms, brown represents C atoms, pink represents H atoms, yellow represents S atoms, blue represents N atoms, purple represents P atoms, and green balls represent B atoms.

Table S1. EXAFS fitting parameters at the Ni K-edge for various samples. (S_0^2 = 0.75).

Sample	Path	CN	R(Å)	σ^2 ($Å^2$)	R factor
	Ni-N	3.83	2.00±0.043	0.015	
R-Ni_{SA}/Ni_{NP}-NSCNT	Ni-S	0.35	2.21±0.024	0.00005	0.01
	Ni-Ni	1.75	2.49±0.021	0.005	
	Ni-N	3.89	1.95±0.019	0.0002	0.013
Ni_{SA}/Ni_{NP}-NCNT	Ni-Ni	2.0	2.42±0.016	0.0047	
	Ni-N	3.25	2.04±0.040	0.005	
Ni_{SA}/Ni_{NP}-NPCNT	Ni-P	0.94	2.29±0.030	0.0014	0.018
	Ni-Ni	3.87	2.52±0.019	0.012	
	Ni-N	3.25	1.88±0.047	0.019	
Ni_{SA}/Ni_{NP}-NBCNT	Ni-B	0.94	1.61±0.029	0.0025	0.02
	Ni-Ni	3.67	2.47±0.012	0.001	

CN: coordination number; R: bond distance; σ^2: Debye-Waller factor; R factor: goodness of fit. $S0^2$ is the amplitude reduction factor, and is set to 0.75 here, according to the experimental EXAFS fitting of Ni foil reference by fixing CN as the known crystallographic value.

Table S2. List of previously reported catalysts for hydrogen peroxide (H_2O_2) production.

Catalyst	H_2O_2 Selectivity	Loading amount	Operating conditions (electrolyte, etc.)	Ref.
Ni_{SA}/Ni_{NP}-NSCNT	92.7~98.8% 0.2 to 0.6 V vs. RHE	0.2 mg cm^{-2}	0.1M KOH	This work
OCG800	92~100% 0.05 to 0.7 V vs.RHE	0.78 mg cm^{-2}	H-cell, 0.1 M KOH + 10 mM EDTA, 0.4 V vs.RHE	[6]
O-CNTs	90 % at 0.65 V (vs RHE)	0.5 mg cm^{-2}	0.1M KOH	[7]

Co_1-NG(O)	82%	10 μg cm^{-2}	0.1M KOH	[8]
Co-SAs/CN	76 % at 0.65 V vs.RHE	/	0.1M KOH	[9]
Pt-SA/rGO	60 % at 0.4 V vs.RHE	101.8 μg cm^{-2}	0.1M KOH	[10]
Ni-SA/G-0	~100 % at 0.4 V vs.RHE	50.8 μg cm^{-2}	0.1M KOH	[11]
CB-Plasma	-100 %	16 μg cm^{-2}	H-Cell,0.1M KOH	[12]
OCB120-CTAB	95.2 % at 0.5 V vs.RHE	/	0.1M KOH	[13]
Br-Ni MOF	88.6% at 0.4 V vs.RHE	50ul(73mg/L)	0.1M KOH	[14]
COF-366-Co	90%	5ug/cm^{-2}	0.1M KOH	[15]
Meso-C	80% at 0.4 V vs.RHE	0.51mg/cm^{-2}	0.1M KOH	[16]

References

[1] L. Kong, M. Wang, Y. Tuo, S. Zhou, J. Wang, G. Liu, X. Cui, J. Wang, L. Jiang, *J. Energy Chem.* **2024**, 88, 183.

[2] a) G. Kresse, J. Furthmüller, *Comp. Mater. Sci.* **1996**, 6, 15; b) G. Kresse , J. Furthmüller *Phys. Rev. B.* **1996**, 54, 11169.

[3] J. P. Perdew, K. Burke, M. Ernzerhof, *Phys. Rev. Lett.* **1996**, 77, 3865.

[4] J. P. Perdew, M. Ernzerhof, K. Burke, *J. Chem. Phys.* **1996**, 105, 9982.

[5] S. Grimme, J. Comput. *Chem.* **2006**, 27, 1787.

[6] K. Lee, J. Lim, M. J. Lee, K. Ryu, H. Lee, J. Y. Kim, H. Ju, H.-S. Cho, B.-H. Kim, M. C. Hatzell, J. Kang, S. W. Lee, *Energy Environ. Sci.* **2022**, 15, 2858.

[7] Z. Lu, G. Chen, S. Siahrostami, Z. Chen, K. Liu, J. Xie, L. Liao, T. Wu, D. Lin, Y. Liu, T. F. Jaramillo, J. K. Nørskov, Y. Cui, *Nat. Catal.* **2018**, 1, 156.

[8] E. Jung, H. Shin, W. Hooch Antink, Y.-E. Sung, T. Hyeon, *ACS Energy Lett.* **2020**, 5, 1881.

[9] H. Xu, S. B. Zhang, J. Geng, G. Z. Wang, H. M. Zhang, *Inorg. Chem. Front.* **2021**, 8, 2829.

[10] X. Song, N. Li, H. Zhang, H. Wang, L. Wang, Z. Bian, *J. Power Sources.* **2019**, 435, 226771.

[11] X. Song, N. Li, H. Zhang, L. Wang, Y. Yan, H. Wang, L. Wang, Z. Bian, *ACS Appl. Mater. Inter.* **2020**, 12, 17519.

[12] Z. Wang, Q.-K. Li, C. Zhang, Z. Cheng, W. Chen, E. A. McHugh, R. A. Carter, B. I. Yakobson, J. M. Tour, *ACS Catal.* **2021**, 11, 2454.

[13] K.-H. Wu, D. Wang, X. Lu, X. Zhang, Z. Xie, Y. Liu, B.-J. Su, J.-M. Chen, D.-S. Su, W. Qi, S. Guo, *Chem.* **2020**, 6, 1443.

[14] M. Liu, Y. Li, Z. Qi, H. Su, W. Cheng, W. Zhou, H. Zhang, X. Sun, X. Zhang, Y. Xu, Y. Jiang, Q. Liu, S. Wei, *J. Phys. Chem. Lett.* **2021**, 12, 8706.

[15] C. Liu, H. Li, F. Liu, J. Chen, Z. Yu, Z. Yuan, C. Wang, H. Zheng, G. Henkelman, L. Wei, Y. Chen, *J. Am. Chem. Soc.* **2020**, 142, 21861.

[16] S. Chen, Z. Chen, S. Siahrostami, T. R. Kim, D. Nordlund, D. Sokaras, S. Nowak, J. W. F. To, D. Higgins, R. Sinclair, J. K. Nørskov, T. F. Jaramillo, Z. Bao, *ACS Sustain. Chem. Eng.* **2018**, 6, 311.
